# Mapping the knowledge of omics in myocardial infarction: A scientometric analysis in R Studio, VOSviewer, Citespace, and SciMAT

**DOI:** 10.1097/MD.0000000000041368

**Published:** 2025-02-14

**Authors:** Xuan Wei, Min Wang, Shengnan Yu, Zhengqi Han, Chang Li, Yue Zhong, Mengzhou Zhang, Tiantong Yang

**Affiliations:** a Key Laboratory of Evidence Science, China University of Political Science and Law, Ministry of Education, Beijing, China; b Institute of Evidence Law and Forensic Science, China University of Political Science and Law, Beijing, China; c Institute for Digital Technology and Law (IDTL), China University of Political Science and Law, Beijing, China; d CUPL Scientometrics and Evaluation Center of Rule of Law, China University of Political Science and Law, Beijing, China.

**Keywords:** Citespace, myocardial infarction, omics research, R Studio, scientometric analysis, Scimat, Vosviewer

## Abstract

Many researchers nowadays choose multi-omics techniques for myocardial infarction studies. However, there’s yet to be a review article integrating myocardial infarction multi-omics. Hence, this study adopts the popular bibliometrics. Based on its principles, we use software like R Studio, Vosviewer, Citespace, and SciMAT to analyze literature data of myocardial infarction omics research (1991–2022) from Web of Science. By extracting key information and calculating weights, we conduct analyses from 4 aspects: Collaboration Network Analysis, Co-word Analysis, Citing and Cited Journal Analysis, and Co-citation and Clustering Analysis, aiming to understand the field’s cooperation, research topic evolution, and knowledge flow. The results show that myocardial infarction omics research is still in its early stage with limited international cooperation. In terms of knowledge flow, there’s no significant difference within the discipline, but non-biomedical disciplines have joined, indicating an interdisciplinary integration trend. In the overall research field, genomics remains the main topic with many breakthroughs identifying susceptibility sites. Meanwhile, other omics fields like lipidomics and proteomics are also progressing, clarifying the pathogenesis. The cooperation details in this article enable researchers to connect with others, facilitating their research. The evolution trend of subject terms helps them set goals and directions, quickly grasp the development context, and read relevant literature. Journal analysis offers submission suggestions, and the analysis of research base and frontier provides references for the research’s future development.

## 1. Introduction

Myocardial infarction is a condition caused by coronary artery stenosis or occlusion, leading to myocardial ischemia, hypoxia, necrosis, and other related pathological diseases. In clinical practice, myocardial infarction is typically diagnosed by detecting changes in cardiac troponin levels.^[1–3]^ It is estimated that approximately 1.8 million people worldwide die of myocardial infarction, the patients who died of myocardial infarction due to the presence of other causes of death attributed to them, so the actual number of deaths due to myocardial infarction may be much higher than the reported figures. Based on this high incidence, myocardial infarction has become a global problem, different countries and regions are facing this medical pressure to different degrees.^[4,5]^

In long term studies, several scholars have elaborated different features of the etiology, pathogenesis, and differential diagnosis of myocardial infarction from the perspectives of genetic inheritance, protein transcription and translation, and biological metabolism. However, there are multiple mechanisms contribute to the development of this disease, a comprehensive understanding of its complex nature cannot be achieved through independent study alone. Research on omics offers a systematic approach that integrates various epidemiological studies such as genomics, proteomics, and metabolomics, aiming at provide a holistic understanding of the disease. Additionally, multi-omics research has achieved to comprehensively evaluate diseases by integrating high-throughput bio-information from different sources.^[6,7]^ By establishing internal relationships among different epidemiological studies, “omics cascade” analysis enables a more comprehensive and systematic understanding of the characteristics of relevant diseases in the biomedical field, especially when combined with machine learning and other artificial intelligence (AI) algorithms. On the one hand, it allows researchers to predict the diagnosis and prognosis of future diseases based on existing bio-information. On the other hand, the improvement of bioinformatics processing capability brought by AI can present a full picture of the target disease and better achieve personalized interventions and treatments.^[8]^ In recent years, researchers are actively using omics methods to study the occurrence and outcome of myocardial infarction and have made some progress in different fields.^[9–11]^

Based on the principles of bibliometrics, this paper employs bibliometrics visualization tools to construct a knowledge mapping of myocardial infarction omics. The study encompasses various major bibliometric aspects, such as collaboration networks, word co-occurrence analysis, citing and cited journals, co-citation analysis, and clustering analysis. These analyses offer insights into current collaborations among countries, institutions, and authors; identify crucial literature contributing to the field’s development or of interest to researchers; examine research directions and their historical evolution; explore knowledge flow patterns; and provide considerations for journal selection. The knowledge structure depicted in this study serves as the foundation for further research while highlighting future frontiers.

By employing visual representations of knowledge, this paper presents the origin, evolution, current status, and future development direction within myocardial infarction omics. It not only provides a comprehensive knowledge mapping for researchers in this field but also facilitates an accessible rapid understanding of research status and acquisition methods. Furthermore, it assists researchers in determining content and direction for future studies while aiding them in selecting appropriate journals to publish their relevant research outcomes.

## 2. Methods

### 2.1. Study design

Bibliometrics, a quantitative research method for analyzing literature information, was first proposed by Pritchard.^[12]^ By conducting statistical analysis of literature, bibliometric method enables the assessment of literature quality, impact, and citations. With the advancements in literature databases, numerous scholars have utilized bibliometrics to construct knowledge structures of multiple research fields, thereby to gain precise insights into current states and future trends of the fields.

By employing bibliometrics, researchers can examine the research strength of a country in a specific field, the development trajectory of research topics, and the comparison of academic quality of different institutions and authors.^[13]^ The popularity of bibliometrics stems from its ability to analyze literature data objectively and effectively through uncovering correlations among various literature information such as journals, authors, countries, citations, keywords, titles, and institutions. Notably, the appearance of diverse scientific visualization software for bibliometrics not only ensures the objectivity of the results but also makes the results more intuitive.

**Figure 1.**
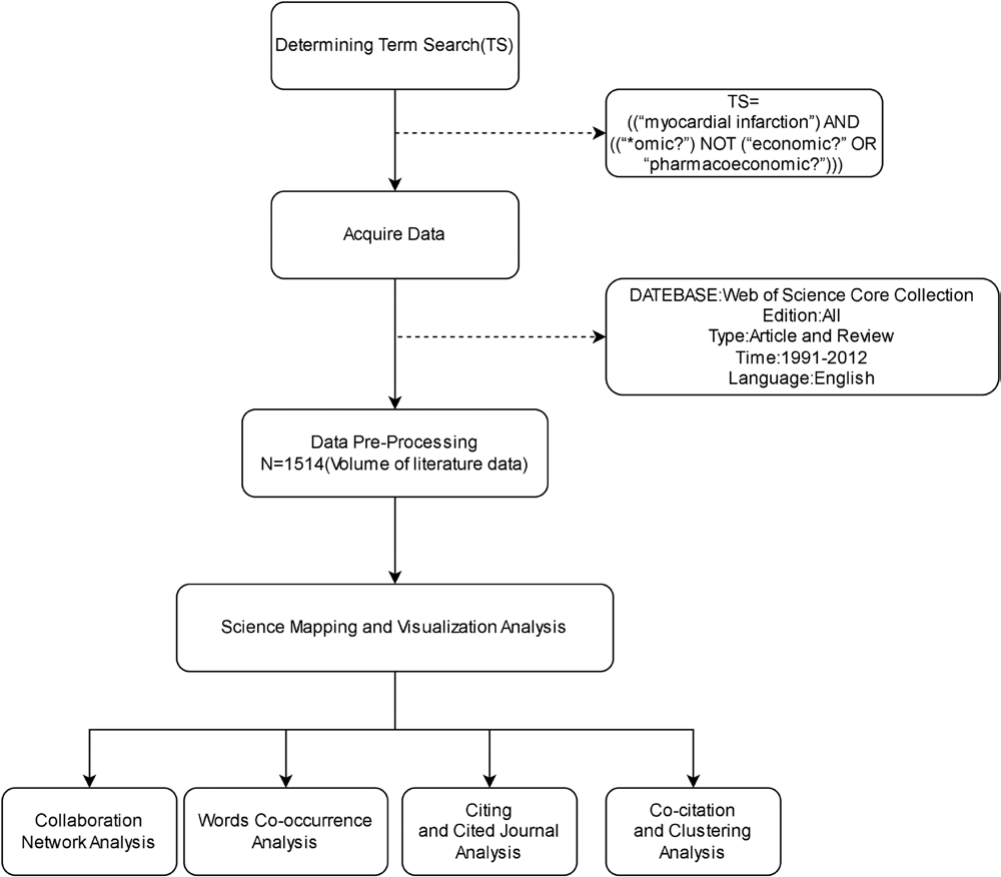
The workflow of science mapping and visualization.

In this paper, several visualization tools were employed to analyze literature data such as Collaboration Network and Word co-occurrence and present the status and trends of myocardial infarction histology research in the form of knowledge maps to provide data support for follow-up studies (Fig. 1).

### 2.2. Acquire and clean data

In this study, the *Web of Science Core Collection* (*WoSCC*) was selected as the literature database. Recognized as one of the most influential databases, *WoSCC* contains a comprehensive collection of scientific research literature. It is widely regarded by researchers as the most suitable databases for conducting bibliometric analyses. Considering the interdisciplinary nature of this research, which encompasses fields such as medicine, biology, and informatics, *WoSCC* is an appropriate data source.^[14]^ Additionally, *WoSCC* employs a rigorous screening mechanism, based on Bradford’s law in bibliometrics. *WoSCC* aggregates influential academic papers across various disciplines, ensuring that the included literature is authoritative and accurately reflects the state of research in the relevant research area.

To achieve the objectives of this study, Boolean rules were applied to establish the following search strategy: TS= ((“myocardial infraction”) AND (“*omic?” OR “pathomic?”) NOT (“economic?” OR “pharmacoeconomic?”)). The article type was limited to *articles* and *reviews*, and the language was restricted to *English*. The time span ranged from 1991, when the first article published, until 2022. Utilizing the classification function of the Web of Science database, the literature data underwent a preprocessing by eliminating documents that were not relevant to the research topic. Ultimately, a total of 1514 relevant documents were obtained.

### 2.3. Statistical analysis

Bibliometric analysis and knowledge mapping were conducted using the Bibliometrix packages in R Studio (4.2.2), Vosviewer (1.6.19), Citespace (6.1.R6), and Scimat (1.1.04). The results were rigorously interpreted according to bibliometric principles.

#### 2.3.1. Collaboration network analysis

Research collaboration is a common situation in scientific research and is one of the indicators of scientific production. In bibliometrics, a network of relationships illustrating collaborations between different research projects is referred to as a collaboration network. The collaboration network not only reflects the productive competence of each research group but also visually displays collaboration trends and modes in the field.^[15]^ In this paper, the indicator “Total link strength” is used to measure the cooperative relationship, indicating the closeness of collaboration based on the number of research groups and depth of research subjects.^[16]^ The paper focuses on 3 types of collaboration networks: author collaboration networks, institutional collaboration networks, and national collaboration networks.

#### 2.3.2. Co-word analysis

High-frequency co-occurrences of words can indicate strong correlations between them. Co-word analysis is employed to discover linkages among subjects in a research field based on the co-occurrence of word pairs or phrases.^[17]^ By constructing a co-word network consisting of related subject terms, co-word analysis succinctly maps the knowledge structure and traces the development of the field of study.^[18,19]^

#### 2.3.3. Citing and cited journal analysis

Journal analysis is an important aspect of bibliometrics as it reflects the output and academic value of scholars in a specific research field. Journals, as carriers of scientific research achievements, provide insights into the distribution in multiple disciplines. By examining both cited and citing journals, it is possible to identify the direction of knowledge flow in terms of research bases and frontiers.

#### 2.3.4. Co-citation and clustering analysis

In co-citation analysis, two documents exhibit a co-citation relationship when they appear in the same reference list of another document. The frequency of citations indicates the influence of a literature in the subject. Frequent co-citations suggest high similarities in the research direction among the cited works. Then, through clustering analysis, clusters with distinct subjects can be identified. Like the co-word analyses, co-citation analysis reveals the knowledge structure of the field and provides a more precise understanding of the overall relationships among the literature. Furthermore, it illustrates the relationship between research bases and frontiers with the help of clustering analysis.^[20,21]^

## 3. Results

### 3.1. The overview of production

**Figure 2.**
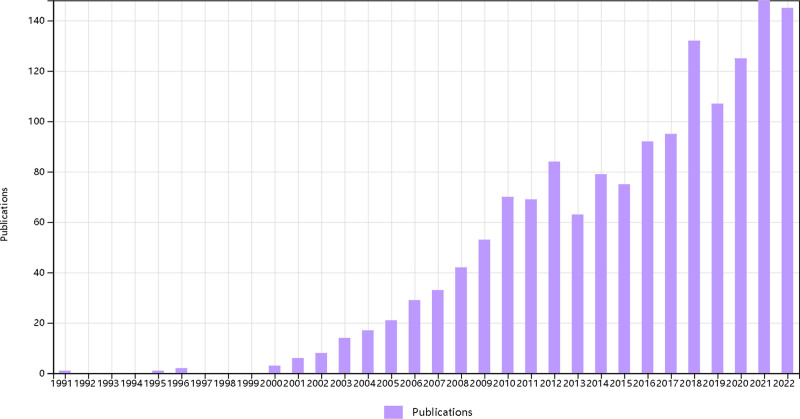
The overview of production.

One of the most fundamental indicators of scientific research output is the number of published papers, which directly reflects the development of a discipline. Results show that the number of published papers in the field of myocardial infarction starts off low in the early stages but increases significantly later. In 2021, a total of 148 papers were published, and in the following year (by the time of data collection of this research) 145 papers had been published. Generally, the number of published papers tends to increase over time, but there are a few instances when it is lower than the previous year. From 2001 to 2010, the number of published papers increased consistently with a relatively flat growth trend. After 2010, the growth became unstable, with a sudden increase in 2018 (Fig. 2).

### 3.2. Collaboration network analysis

**Figure 3.**
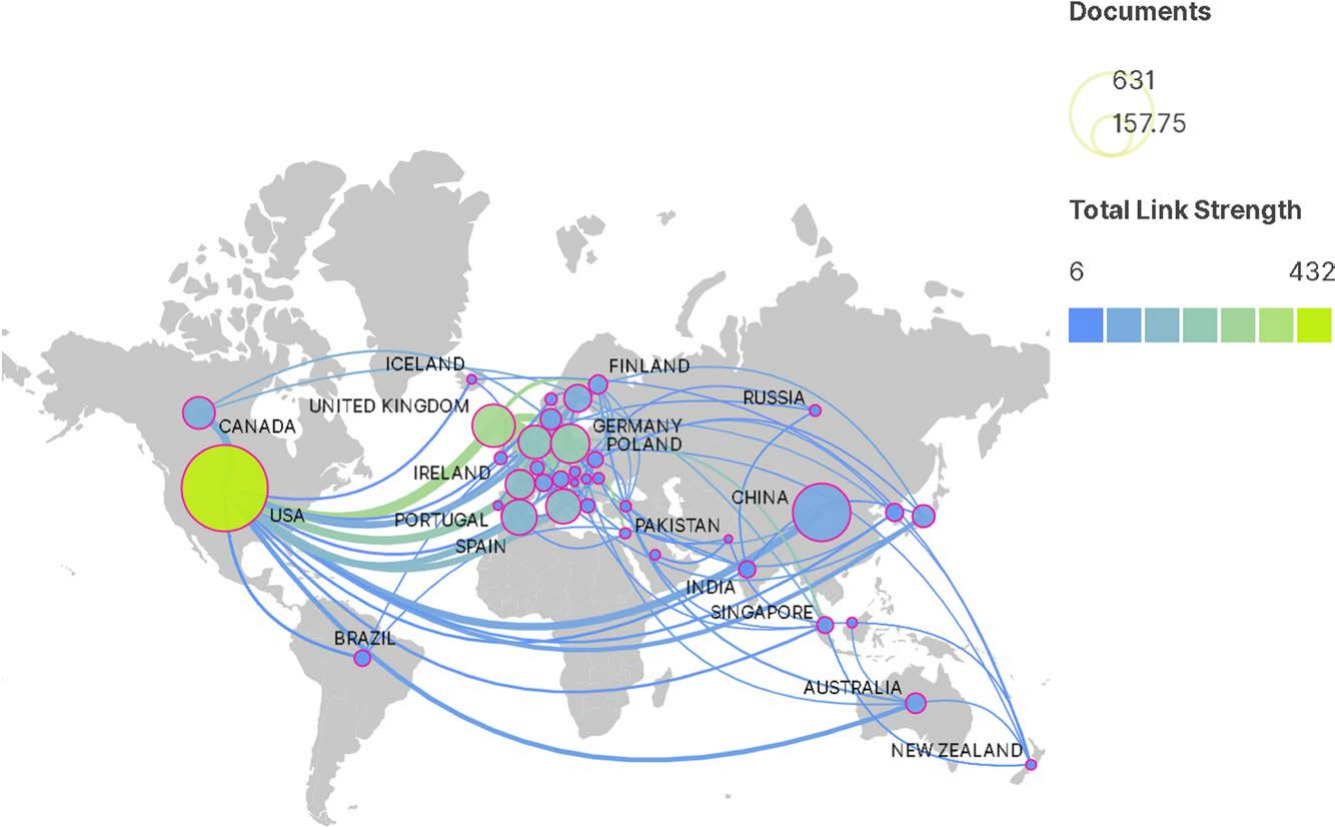
The collaboration network of countries.

**Figure 4.**
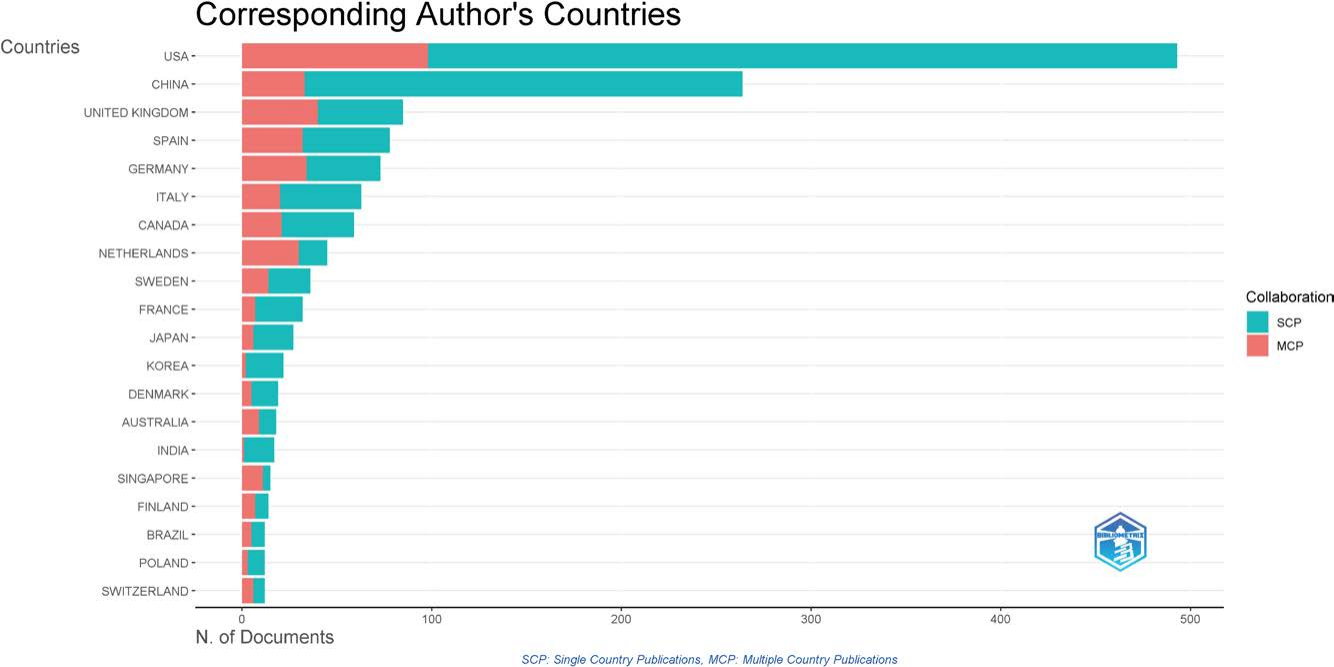
The relation of corresponding author’s countries.

In the field of myocardial infarction omics, the top 5 countries with the highest number of publications were the United States (631), China (285), the United Kingdom (156), Germany (129), and Spain (107). Regarding the strength of inter-country cooperation, the United States, the United Kingdom, Germany, Iceland, and Italy emerged as the top 5 countries. The results indicate that the United States ranked first in terms of both the number of articles published and the strength of cooperation in this field. China, on the other hand, ranked second in the number of articles published but did not lead the cooperation strength list. This is because research in China is primarily represented by domestic authors, with limited international co-authorship, resulting in lower cooperation strength. Overall, apart from the United States, other countries exhibit a relatively low overall number of publications and a small proportion of transnational coauthors in their total publications, indicating relatively weak cooperation relationships (Figs. 3 and 4).

**Figure 5.**
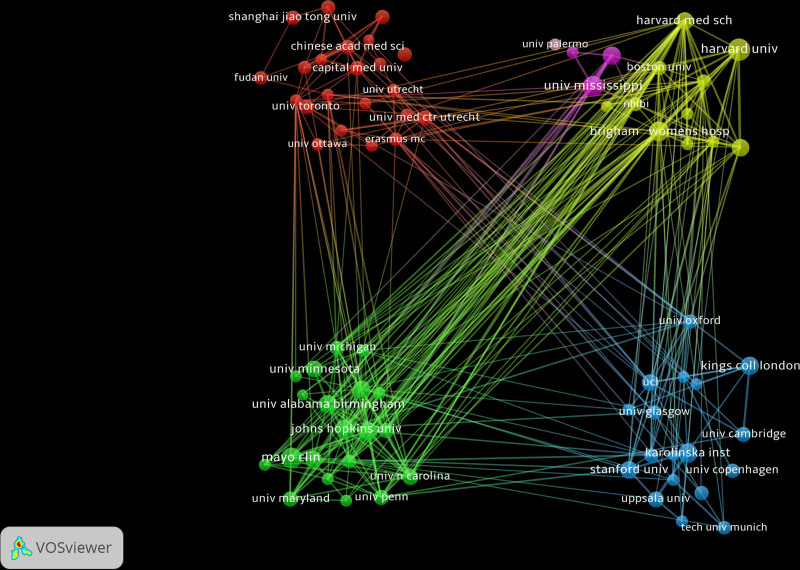
The collaboration network of institutions.

Research institutions are mainly stable academic organizations, which usually contribute consistent academic outputs and have organized collaboration and exchange systems. Therefore, collaboration among institutions is generally more flexible compared with collaboration among nations, and more organized compared with collaboration among individual authors. The results show that Harvard University produced the most publications, with 40 papers and a citation count of 3550. The University of Mississippi followed with 35 papers, Mayo Clinic and Brigham Women’s Hospital tied with 31 papers each, and Karolinska Institute with 30 papers (Fig. 5).

**Figure 6.**
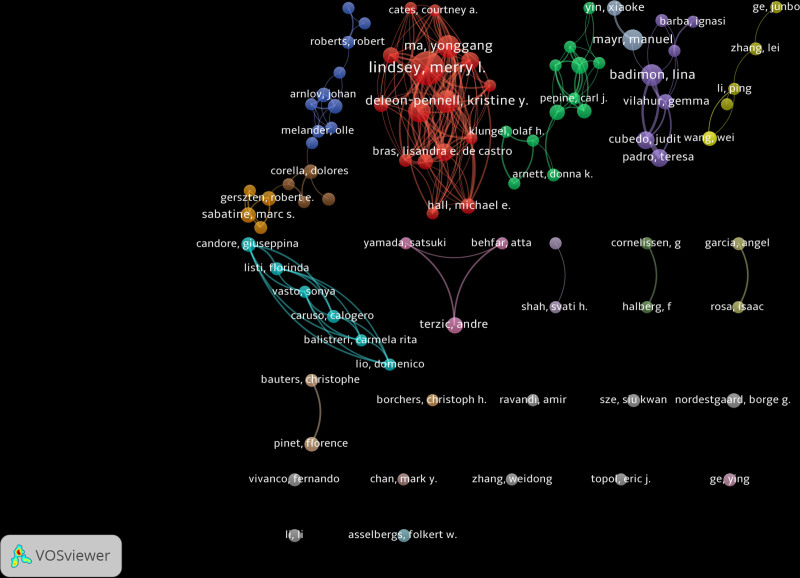
The collaboration network of authors.

**Figure 7.**
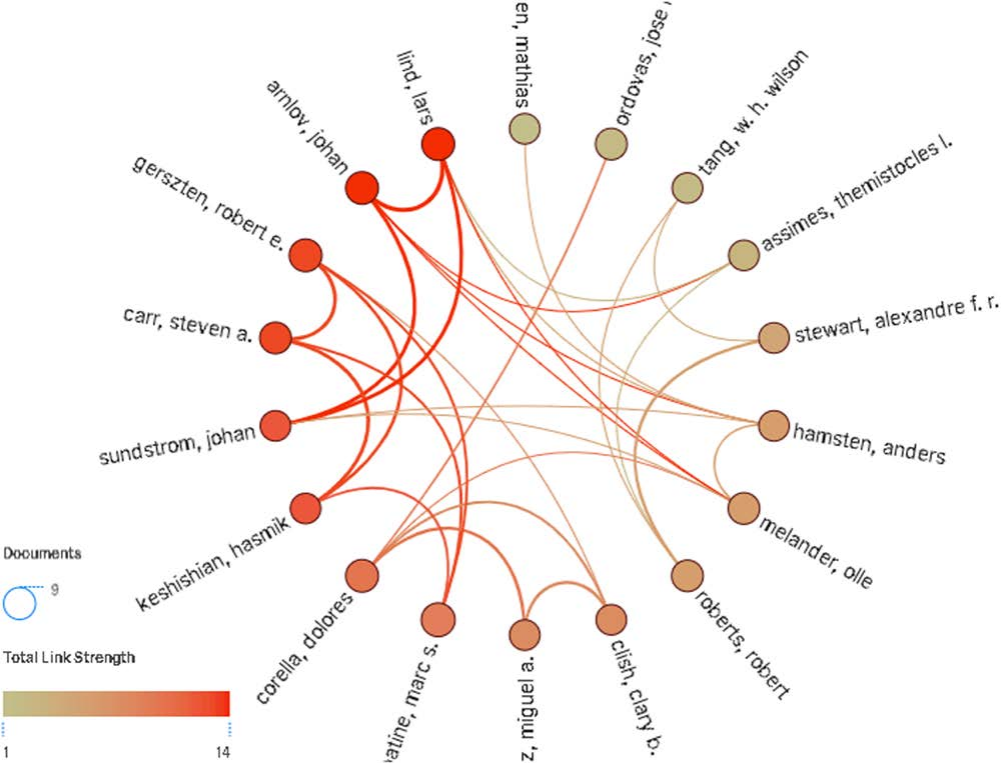
The largest group of the connected authors.

Authors represent the smallest unit of scientific research production and exhibit greater flexibility in scientific research collaboration. The top 5 authors in terms of publication count were Lindsey (40), Iyer (18), Ma (18), Deleon-Pennell (18), and Badimon (16). The top 5 most cited authors were Lindsey (1722 citations), Iyer (1033 citations), Mayr (955 citations), Ma (914 citations), and Deleon-Pennell (795 citations). Additionally, the results reflect a clustering distribution of collaborations. Academic connections lead to formations of distinct academic groups. The largest set of connected authors in the co-authorship network is dominated by Lindsey. This group has a tight collaboration relationship, a higher total citations frequency and a greater academic influence (Figs. 6 and 7).

### 3.3. Words co-occurrence analysis

#### 3.3.1. Overlapping map

Co-word analysis focuses on words extracted from literature, and sources include but not limited to titles, abstracts, and author keywords. The Overlapping map allows us to observe how the relevant keywords in a particular research field change over time. In the Overlapping map, the number inside each circle indicates the number of keywords in the corresponding period. The number between circles represent the retention number and retention rate of the words. A downward arrow indicates the newly added words, while an upward arrow represents the eliminated words.^[22]^

**Figure 8.**
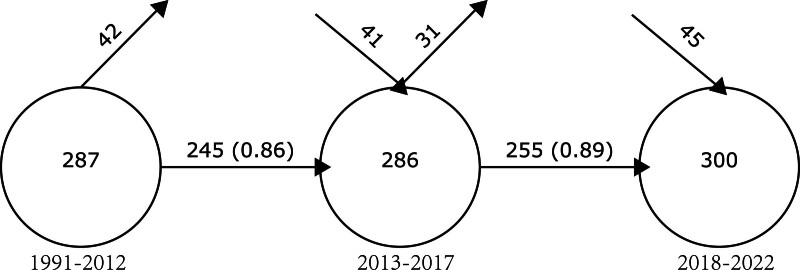
The overlapping map.

This paper divides the period from 1991 to 2022 into 3 sections (1991–2012, 2013–2017, and 2018–2022) based on the number of documents so that each section contains roughly the same number of documents. The number of words in the 3 intervals is 287, 286, and 300, and the number of retained words (retention rate) is 245 (0.86) and 255 (0.89), respectively (Fig. 8).

#### 3.3.2. Co-occurrence analysis of words meaning

In co-word analysis, it is necessary to analyze word meanings and the connections between them to outline the knowledge framework of the field. This paper utilizes standardized terms (index keywords) to express the development of the field in a normalized form.^[23]^

**Figure 9.**
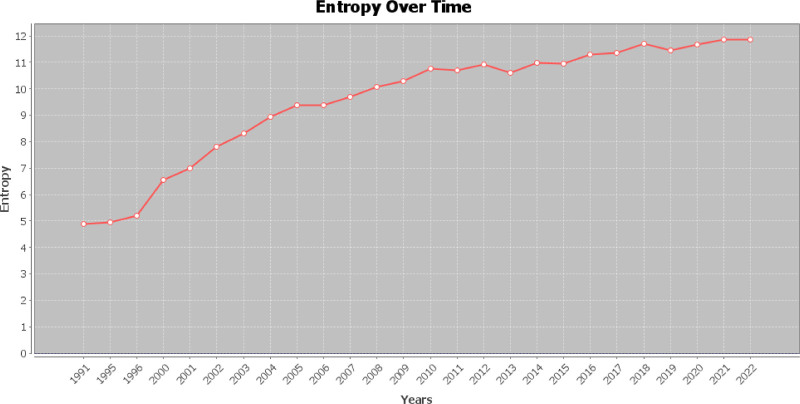
Entropy map.

Terms analysis is essentially a dimensionality reduction analysis of literature, and an entropy map is used to illustrate the entropy change of terms. An increase in entropy indicates that meanings of words become richer. The entropy map reveals the growing variety of terms in the field of myocardial infarction omics research, implying a gradual enrichment of research content (Fig. 9).

**Figure 10.**
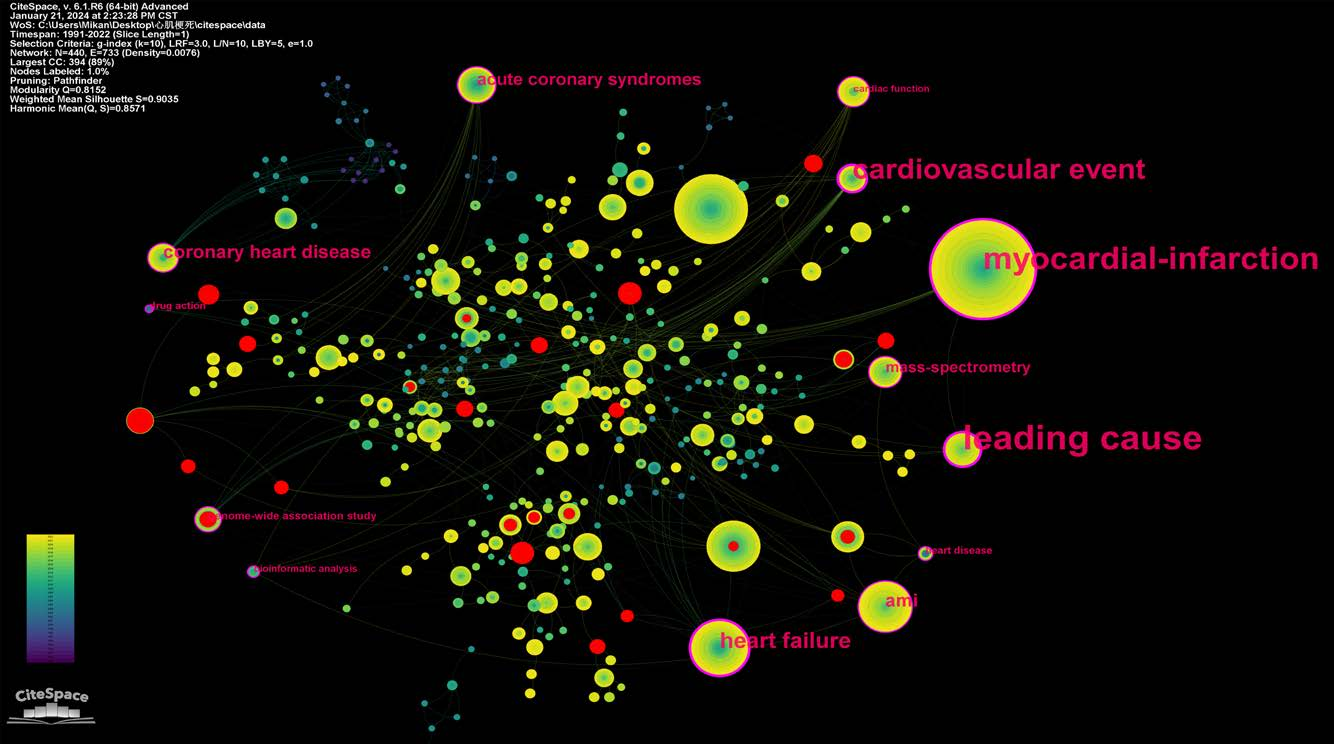
Term co-occurrence network.

**Figure 11.**
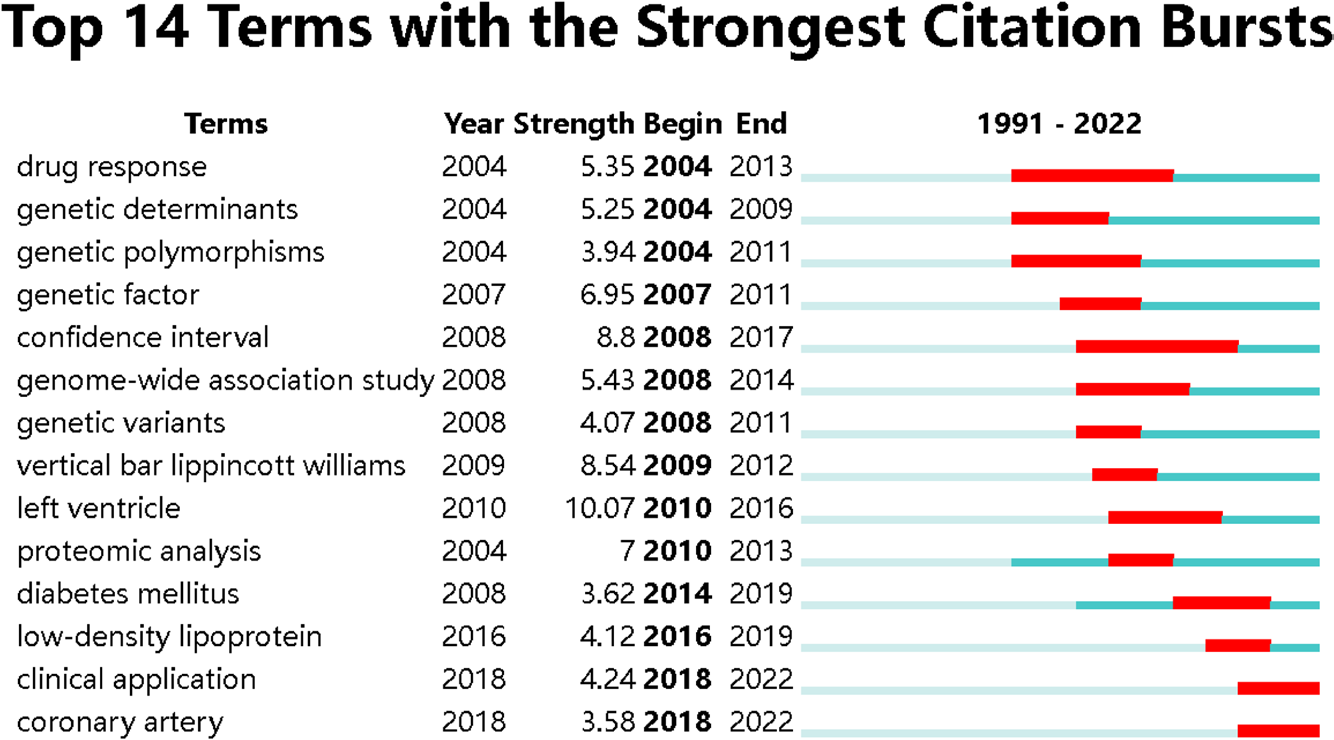
Top 14 burst terms with the strongest citation.

In the term co-occurrence network, the size of a node represents the count, and the lines represent the co-occurrence relation among the terms. The red nodes are burst nodes, while the pink outlined nodes are high centrality nodes. Burst nodes receive additional attention in a short period of time^[24]^ (Fig. 10). Centrality, on the other hand, evaluates the status of a node in the network. Nodes with high centrality occupy key positions in the co-occurrence network of terms, which is generally reflected in the frequent occurrence of the word itself or close connection with other words (Table 1). Figure 11 displays the top 14 words with the highest burst in the term co-occurrence network map.

**Table 1:** Terms clustering, burst terms and centrality nodes

Cluster	Burst terms	Centrality node
#0 extracellular matrix	Left ventricle	
#1 pharmacogenomics	Proteomic analysis	Bioinformatic analysis (0.10) Ami (0.20) Heart disease (0.24) Mass-spectrometry (0.16)
#2 mediterranean diet		Cardiovascular event (0.30)
#3 coronary artery disease	Confidence interval Vertical bar lippincott Williams Low-density lipoprotein Coronary artery	
#4 heart failure	Drug response Genetic determinants Genetic polymorphisms	Drug action (0.11) Heart failure (0.24)
#5 transyear		
#6 gut microbiota		
#7 artificial intelligence	Clinical application	Acute coronary syndromes (0.18)
#8 traditional chinese medicine		Cardiac function (0.10)
#9 genetics	Genetic factor Diabetes mellitus Genome-wide association study genetic variants	Genome-wide-association study (0.12)
#10 myocardial infarction		Myocardial infarction (0.34) Leading cause (0.37)
#11 angiotensin converting enzyme inhibitors		Coronary heart disease (0.20)
#12 percutaneous coronary intervention		
#13 clopidogrel		
#15 clinical trials		
#16 acute myocardial infraction		
#17 stemi		
#19 perilipin		
#20 ldl-cholesterol		

**Figure 12.**
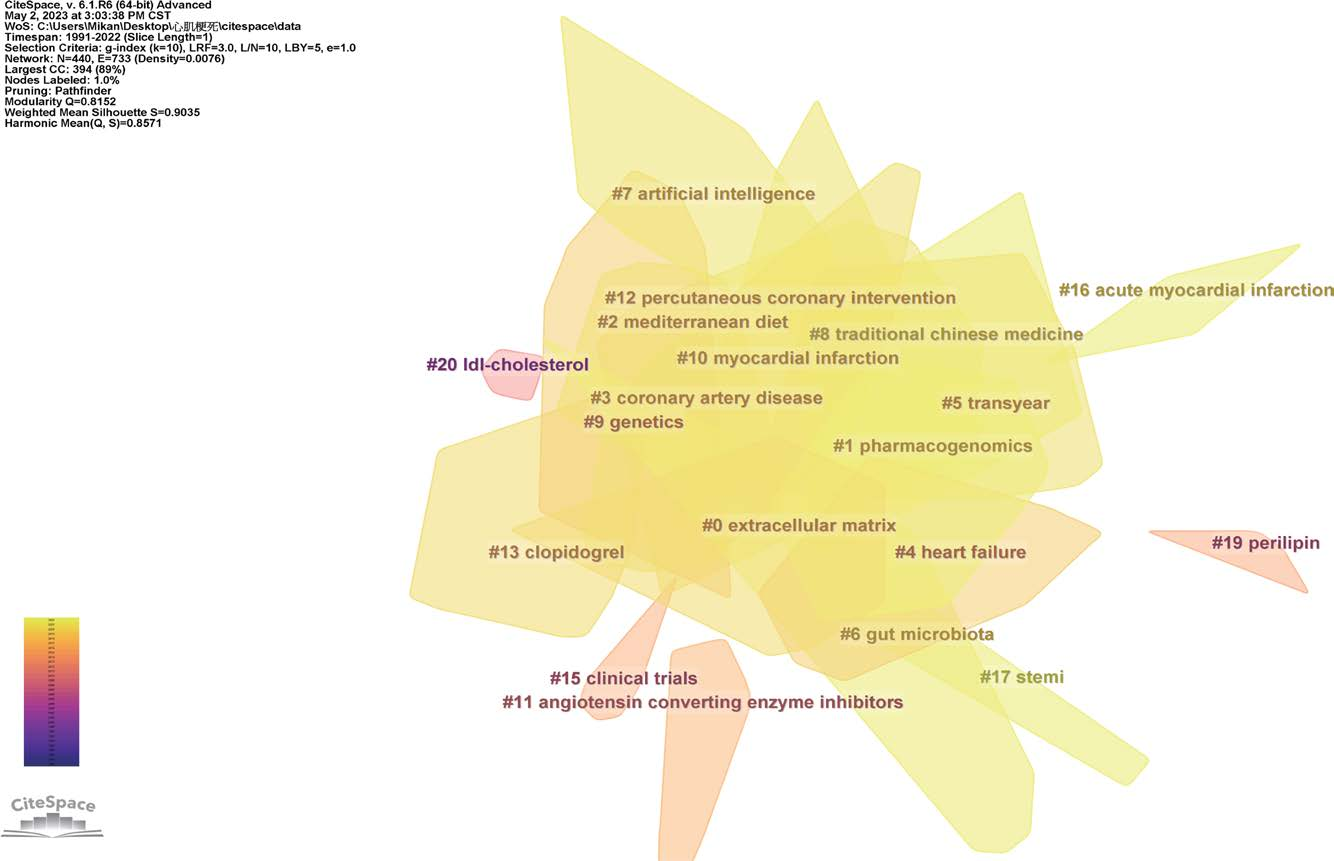
The overview of clustering map.

**Figure 13.**
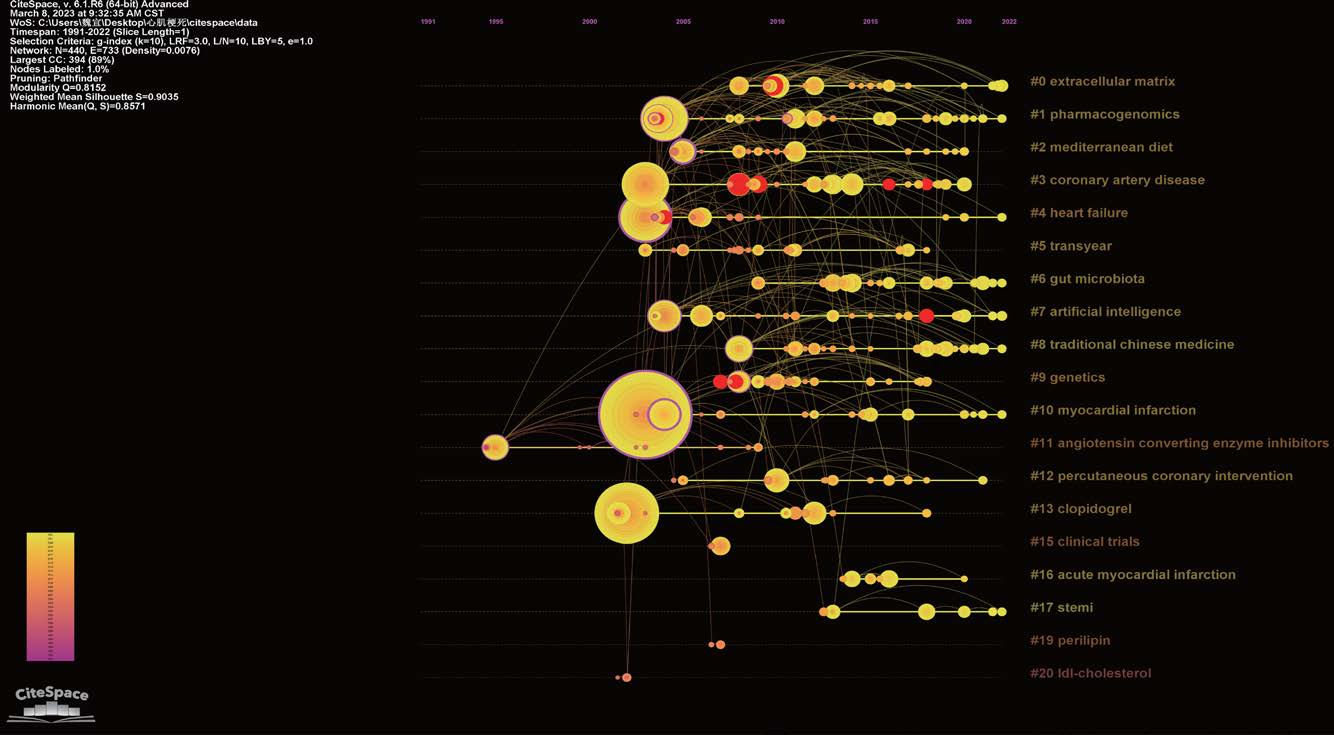
The timeline of clustering map.

The term clustering map and the term timeline map are generated based on the co-occurrence network diagram of themes. (Figs. 12 and 13) The modularity value of the clustering is 0.8152 (>0.3), and the silhouette value is 0.9035 (>0.7), indicating a significant and convincing clustering effect^[25]^ (Table 1).

#### 3.3.3. Strategic map

**Figure 14.**
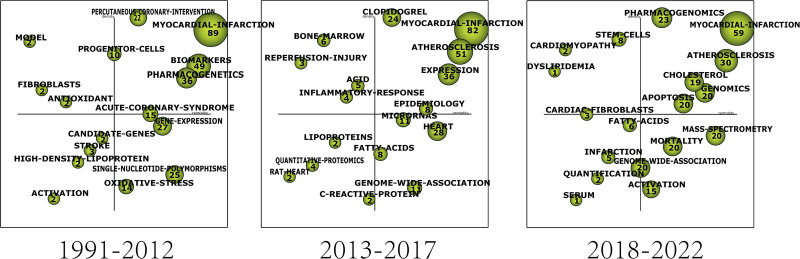
Strategic map.

The strategic map utilizes centrality as the horizontal axis and density as the vertical axis. Centrality is proportional to the degree of correlation between a theme and another, while density is proportional to the inter-cluster correlation of a theme. The nodes represent clusters with different themes, and the node size is proportional to the G-index of theme clustering. Figure 14 represents the status of themes at different stages. When a theme falls in the first quadrant, it is considered as an *engine theme.* If the theme is located in the second quadrant, it is considered as an *isolated* or *specialized theme*. Themes in the third quadrant are considered as *emerging* or *dying themes*, while themes in the fourth quadrant are considered as *innovation themes*.^[26]^

In the strategic map, 16 clusters were formed from 1991 to 2012, 17 clusters were from 2013 to 2017, and 18 were from 2018 to 2022 (Table 2; Fig. 14).

**Table 2:** Term clustering in strategic map

Term clustering	G-index	Quadrant
1991–2012		
MYOCARDIAL-INFARCTION	89	Q1
BIOMARKERS	49	Q1
PHARMACOGENETICS	36	Q1
PERCUTANEOUS-CORONARY-INTERVENTION	22	Q1
PROGENITOR-CELLS	10	Q1/Q2
MODEL	2	Q2
FIBROBLASTS	2	Q2
ANTIOXIDANT	2	Q2
STROKE	3	Q3
CANDIDATE-GENES	2	Q3
HIGH-DENSITY-LIPOPROTEIN	2	Q3
ACTIVATION	2	Q3
GENE-EXPRESSION	27	Q4
SINGLE-NUCLEOTIDE-POLYMORPHISMS	25	Q4
OXIDATIVE-STRESS	14	Q4
ACUTE-CORONARY-SYNDROME	15	Q4/Q1
2013–2017		
MYOCARDIAL-INFARCTION	82	Q1
ATHEROSCLEROSIS	51	Q1
EXPRESSION	36	Q1
CLOPIDOGREL	24	Q1
EPIDEMIOLOGY	8	Q1
BONE-MARROW	6	Q2
ACID	5	Q2
INFLAMMATORY-RESPONSE	4	Q2
REPERFUSION-INJURY	3	Q2
QUANTITATIVE-PROTEOMICS	4	Q3
LIPOPROTEINS	2	Q3
RAT-HEART	2	Q3
C-REACTIVE-PROTEIN	2	Q3
HEART	28	Q4
MICRORNAS	11	Q4
GENOME-WIDE-ASSOCIATION	11	Q4
FATTY-ACIDS	8	Q4
2018–2022		
MYOCARDIAL-INFARCTION	59	Q1
ATHEROSCLEROSIS	30	Q1
PHARMACOGENOMICS	23	Q1
GENOMICS	20	Q1
APOPTOSIS20	20	Q1
CHOLESTEROL	19	Q1
STEM-CELLS	8	Q2
CARDIOMYOPATHY	2	Q2
DYSLIPIDEMIA	1	Q2
CARDIAC-FIBROBLASTS	3	Q2/Q3
FATTY-ACIDS	6	Q3
INFARCTION	5	Q3
QUANTIFICATION	2	Q3
SERUM	1	Q3
GENOME-WIDE-ASSOCIATION	20	Q4/Q1
MASS-SPECTROMETRY	20	Q4
MORTALITY	20	Q4
ACTIVATION	15	Q4

#### 3.3.4. Evolution map

**Figure 15.**
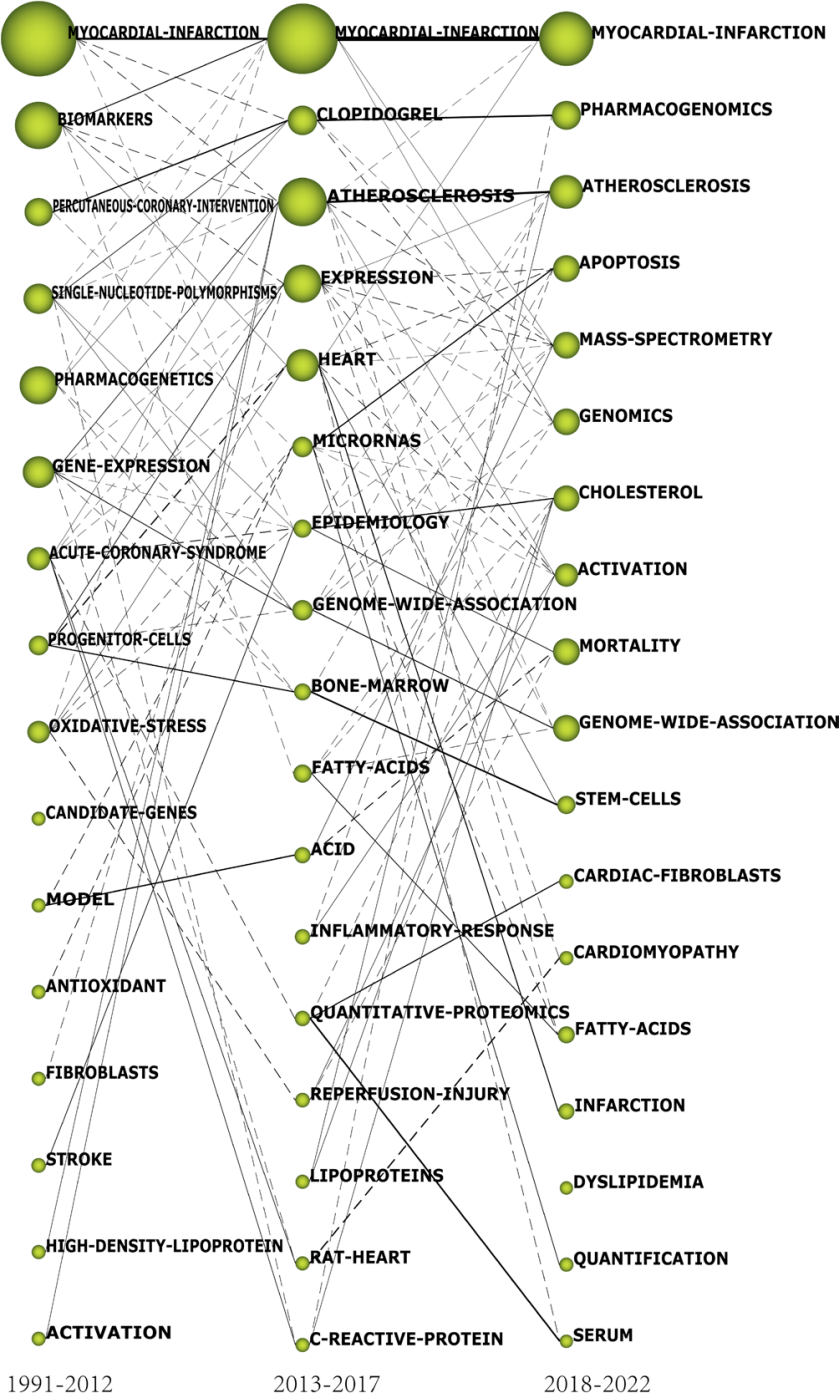
Evolution map.

The evolution map is used to demonstrate the evolutionary pathway of clustered themes. The size of nodes is proportional to the G-index of the relevant terms. Solid lines indicate the 2 nodes share significant keywords, and dashed lines mean the 2 share minor keywords. The thickness of the lines indicates the intensity of sharing.^[22,26]^ The evolutionary paths can be categorized into the research methods, the physiological/pathological states, and the biological structures according to the research content (Fig. 15).

#### 3.3.5. Three-fields plot

**Figure 16.**
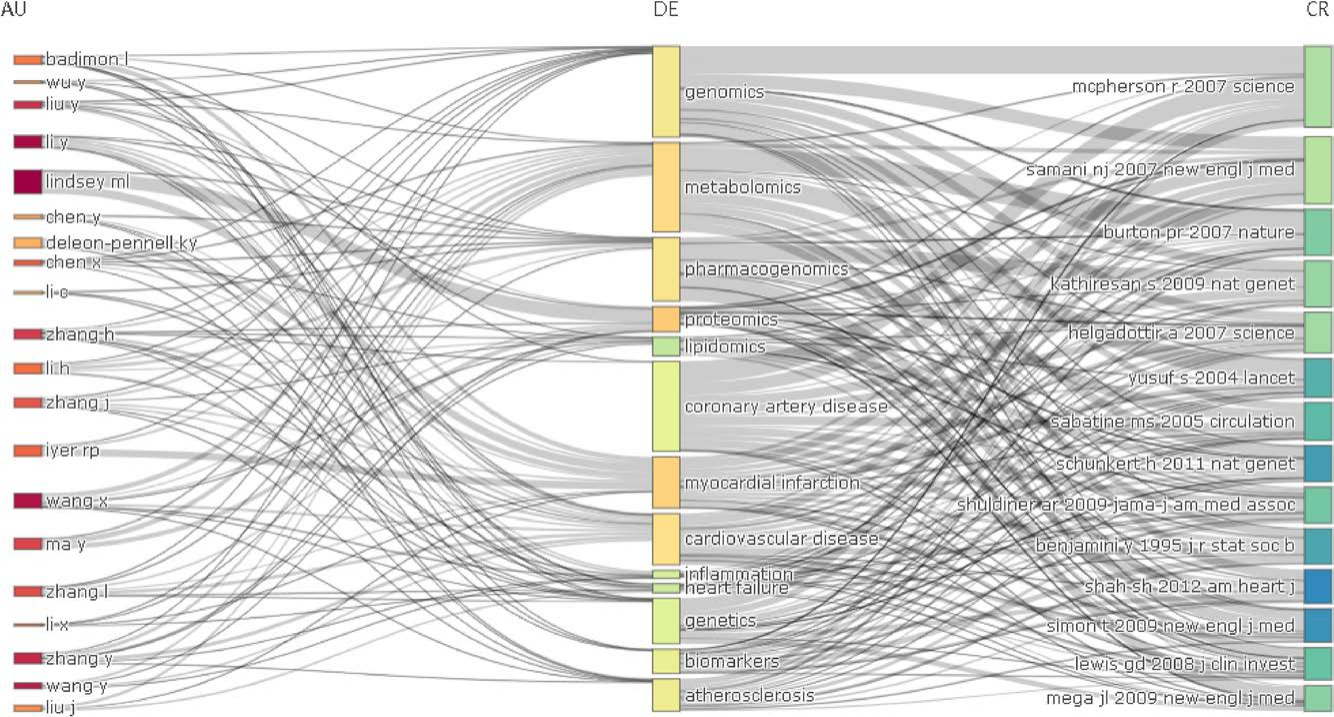
Three fields plot.

The Three-fields Plot is a visualization map commonly used in bibliometrics. It is consisting of 3 different indices chosen based on the purpose. In this paper, *authors*, *keywords*, and *references,* which are closely related to each other, are selected to help researchers better understand the knowledge structure and flow of the research field. Researchers can identify influential authors, major research results, and important research literature by using any index as an entry point and tracking relevant paths (Fig. 16).

### 3.4. Citing and cited journal analysis

**Figure 17.**
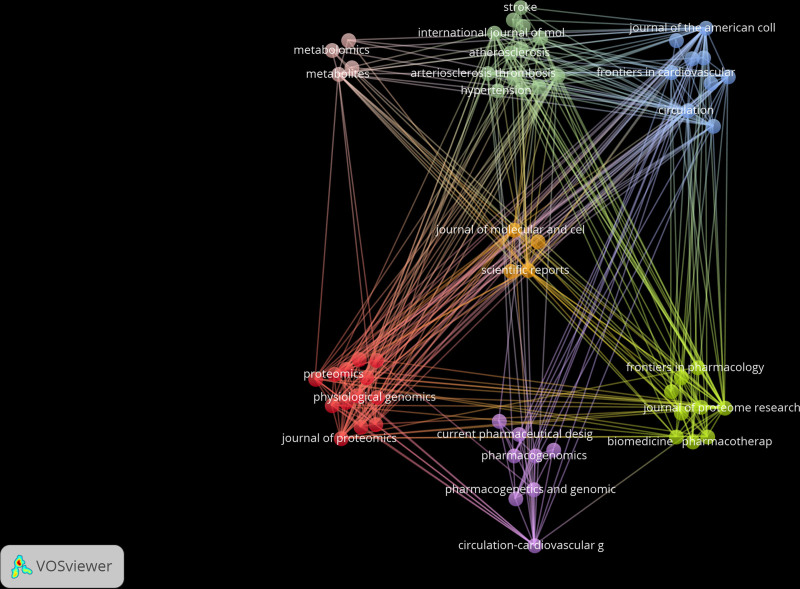
The cluster map of citing journal.

**Figure 18.**
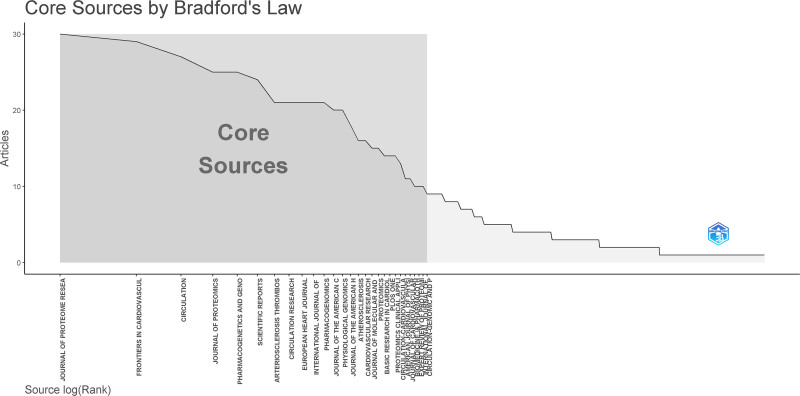
Core journal by Bradford’s law.

Citing journals are typically regarded as leading journals in the field of research. Figure 17 displays the network of the citing journals, while Figure 18 shows the core journals in the field calculated according to Bradford’s Law, highlighting the journals closely related to the field. The top 5 journals are *JOURNAL OF PROTEOME RESEARCH*, *FRONTIERS IN CARDIOVASCULAR MEDICINE*, *CIRCULATION*, *JOURNAL OF PROTEOMICS*, and *PHARMACOGENETICS AND GENOMICS* (Figs. 17 and 18).

**Figure 19.**
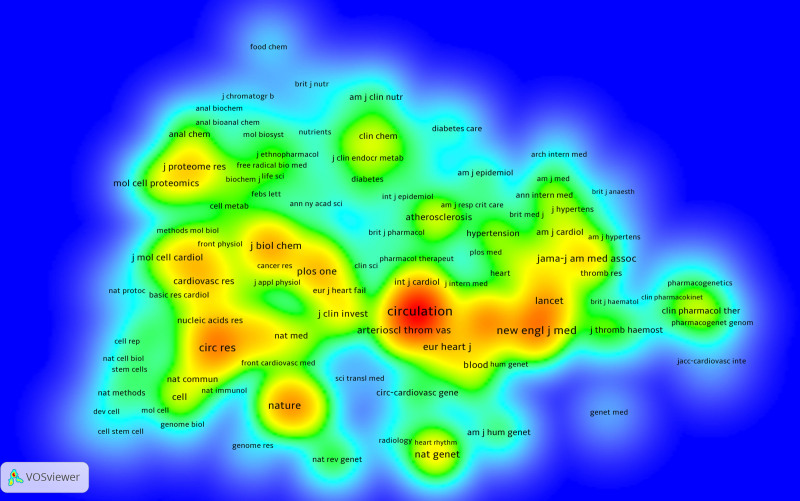
Density map of cited journal.

Cited journals are considered the foundation in the field of research. The density map of cited journals illustrates the popularity of these journals. The redder the region, the more frequently the journal is cited in the field. The top 5 cited journals are *CIRCULATION* (4839), *NEW ENGLAND JOURNAL OF MEDICINE* (2559), *JOURNAL OF THE AMERICAN COLLEGE OF CARDIOLOGY* (2409), *CIRCULATION RESEARCH* (2094), and *NATURE* (1814) (Fig. 19).

**Figure 20.**
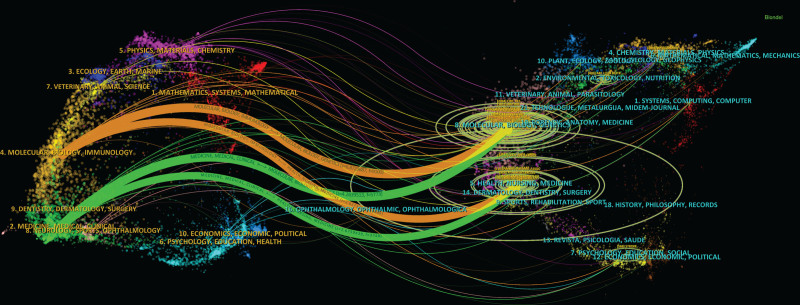
Overlay dual maps.

In the dual-map overlay of journals, citing journals are distributed in the left-hand side, while cited journals are distributed in the right-hand side. According to the map, the 4 main directions are as follows: *molecular, biological, immunological-molecular, biological, genetic*; *molecular, biological, immunology-health, nursing, medicine*; *medical, medical, clinical-molecular, biological, genetic*; and *medicine, medical, clinical-health, nursing, medicine* (Fig. 20).

### 3.5. Co-citation and clustering analysis

**Figure 21.**
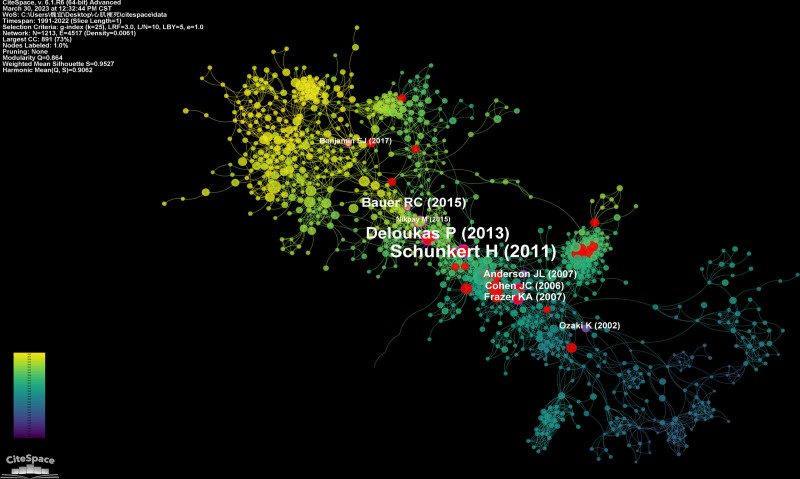
The network of co-citation references.

**Figure 22.**
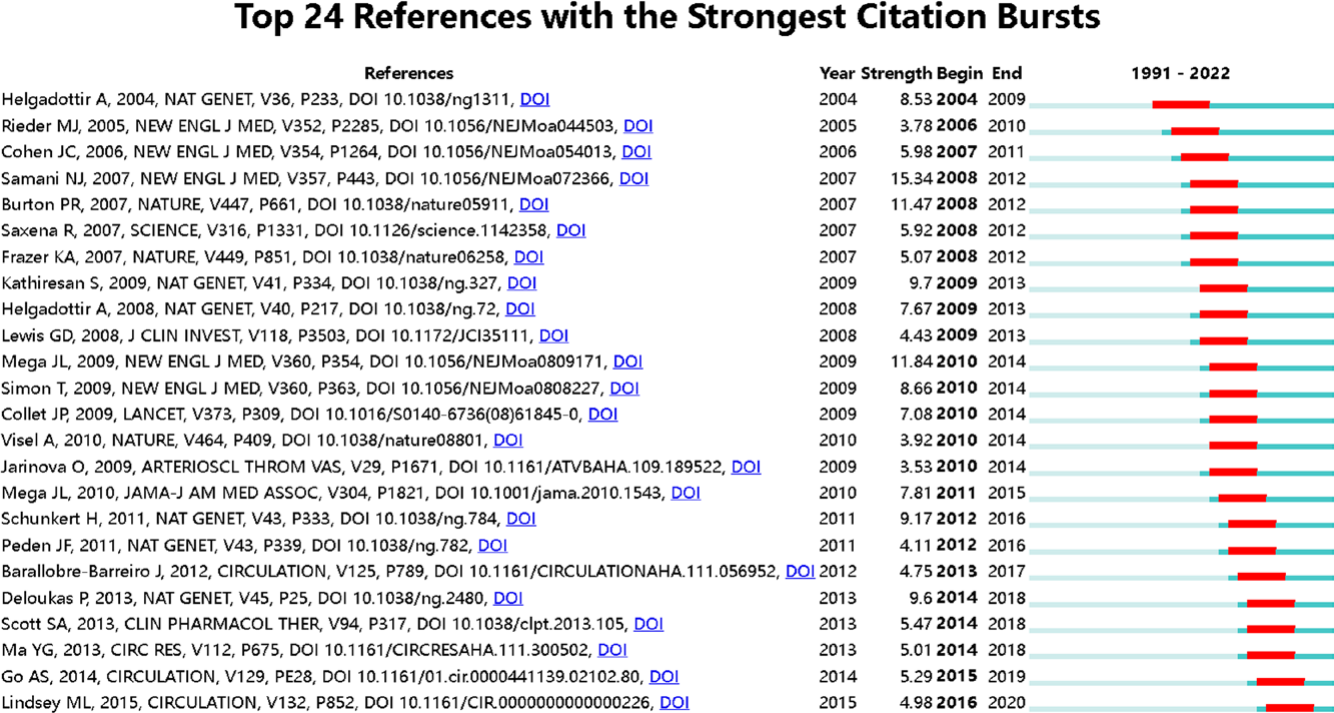
Top 24 references with the strongest citation.

Similar to the term co-occurrence network, the red nodes in the network of co-citation references represent nodes of burst literature, and the pink outlined nodes represent nodes of literature with high centrality. The label in Figure 21 shows the literature whose centrality is greater than 0.10 (Fig. 21). Figure 22 lists the top 24 references with the strongest citation bursts.

**Figure 23.**
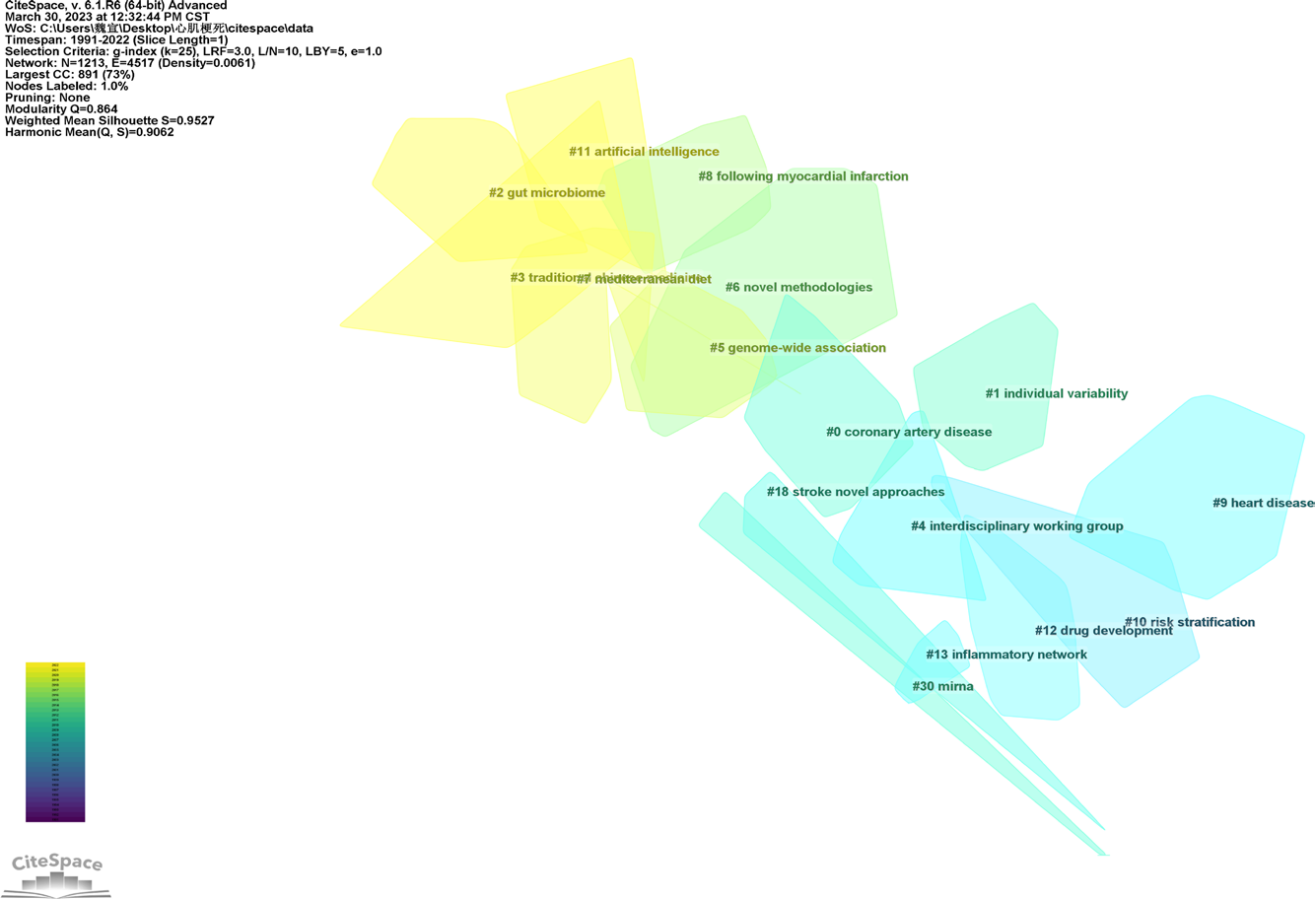
The clustering map of co-citation references.

**Figure 24.**
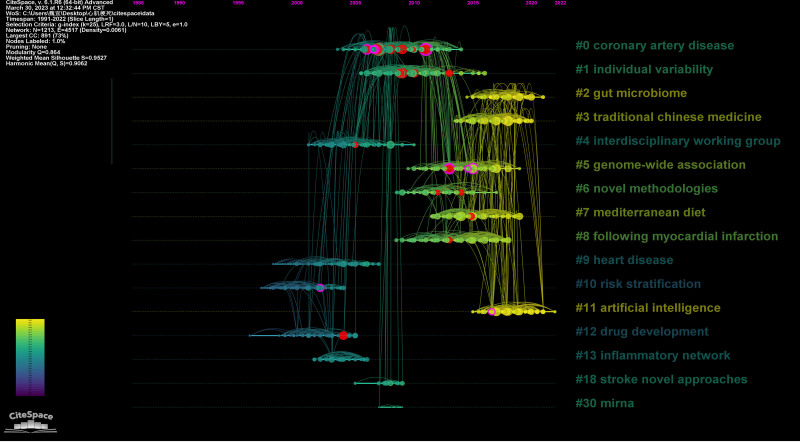
The timeline map of co-citation references.

A total of 16 clusters were obtained after clustering the co-cited references. The modularity value of the clustering is 0.864, which is greater than 0.3, and the silhouette value is 0.9527, which is greater than 0.7, suggesting that the clustering has a good classification effect and credibility (Fig. 23). The timeline map of co-citation reference reflects the distribution of different literature on the time axis according to clustering conditions (Table 3; Fig. 24).

**Table 3:** The clustering information

Cluster ID	Size	Silhouette	Label (LLR)	Average year
0	101	0.927	Coronary artery disease	2008
1	83	0.98	Individual variability	2009
2	70	0.926	Gut microbiome	2018
3	69	0.922	Traditional chinese medicine	2016
4	69	0.951	Interdisciplinary working group	2004
5	66	0.966	Genome-wide association	2014
6	63	0.966	Novel methodologies	2012
7	62	0.938	Mediterranean diet	2015
8	60	0.961	Following myocardial infarction	2013
9	54	0.972	Heart disease	2003
10	54	0.971	Risk stratification	2000
11	52	0.958	Artificial intelligence	2018
12	49	0.94	Drug development	2001
13	26	0.998	inflammatory network	2004
18	9	0.993	Stroke novel approaches	2008
30	4	0.99	Mirna	2008

Based on the distribution of co-cited references in the timeline map of co-citation references, the burst intensity and centrality of nodes, the paper chose *#0 coronary artery disease*, *#1 individual variability*, *#2 gut microbiome*, and *#11 artificial intelligence* to analyze. Among them, clusters 0 and 1 are clusters with more burst and/or high centrality literature, and clusters 2 and 11 are recent popular studies.

The name of Cluster 0 is “Coronary Artery Disease.” The research bases are ranked according to the number of citations in the literature, namely Samani NJ (2007), McPherson R (2007), Helgadottir A (2007), and Kathiresan S (2009). The research frontiers are ranked based on the coverage of the cited references. The larger the coverage, the more the cited references. The top 5 are Musunuru K (2010), Baudhuin LM (2009), Damani SB (2011), Johansen CT (2009), and Roberts R (2014). (Table 4).

**Table 4:** Research base and research frontier of Cluster 0

Cluser#0 coronary artery disease			
Cited references		Citing references	
Freq	Author (year) title	Coverage	Author (year) title
36	Samani NJ (2007) Genomewide Association Analysis of Coronary Artery Disease	25	Musunuru K (2010) Genetics of coronary artery disease
35	McPherson R (2007) Disease A Common Allele on Chromosome 9 Associated with Coronary Heart Disease	23	Baudhuin, LM (2009) Genetics of coronary artery disease: focus on genome-wide association studies
32	Helgadottir A (2007) A Common Variant on Chromosome 9p21 Affects the Risk of Myocardial Infarction	23	Damani, SB (2011) Emerging clinical applications in cardiovascular pharmacogenomics.
27	Burton PR (2007) Genome-wide association study of 14,000 cases of 7 common diseases and 3000 shared controls	22	Johansen, CT (2009) Predictive genetic testing for coronary artery disease
24	Kathiresan S (2009) Genome-wide association of early-onset myocardial infarction with single nucleotide polymorphisms and copy number variants	21	Roberts, R (2014) Genetics of coronary artery disease

The name of Cluster 1 is “Individual Variability,” and the top 5 research bases are Mega JL (2009), Simon T (2009), Shuldiner AR (2009), Mega JL (2010), and Collet JP (2009). The top 5 research frontiers are Xie H (2011), Fisch AS (2013), Yin T (2011), Perry CG (2013), and Cavallari LH (2011) (Table 5).

**Table 5:** Research base and research frontier of cluster 1

#1 individual variability			
Cited references		Citing references	
Freq	Author (year) title	Coverage	Author (year) title
29	Mega JL (2009) Cytochrome *P*-450 Polymorphisms and Response to Clopidogrel	30	Xie, H (2011) Individual variability in the disposition of and response to clopidogrel: pharmacogenomics and beyond
26	Simon T (2009) Genetic Determinants of Response to Clopidogrel and Cardiovascular Events	22	Fisch, AS (2013) Pharmacogenomics of anti-platelet and anti-coagulation therapy
25	Shuldiner AR (2009) Association of Cytochrome P450 2C19 Genotype With the Antiplatelet Effect and Clinical Efficacy of Clopidogrel Therapy	19	Yin, T (2011) Pharmacogenomics of clopidogrel: evidence and perspectives
23	Mega JL (2010) Reduced-Function CYP2C19 Genotype and Risk of Adverse Clinical Outcomes Among Patients Treated With Clopidogrel Predominantly for PCI	18	Perry, CG (2013) Pharmacogenomics of anti-platelet therapy: how much evidence is enough for clinical implementation?
21	Collet JP (2009) Cytochrome P450 2C19 polymorphism in young patients treated with clopidogrel after myocardial infarction: a cohort study	18	Cavallari, LH (2011) Role of cytochrome p450 genotype in the steps toward personalized drug therapy

In Cluster 2, which is titled “Gut Microbiome,” the top 5 research bases are Benjamin EJ (2019), Chong J (2018), Virani SS (2020), Anderson JL (2017), and Tallquist MD (2017). The top 5 research frontiers are Marin Sedeno E (2021), Sayers JR (2021), Dai Z (2021), Xu J (2020), and Qian B (2022) (Table 6).

**Table 6:** Research base and research frontier of cluster 2

#2 Gut microbiome			
Cited references		Citing references	
Freq	Author (year) title	Coverage	Author (year) title
15	Benjamin EJ (2019) Heart Disease and Stroke Statistics— 2019 Update: A Report From the American Heart Association	14	Marin-sedeno, E (2021) Understanding the adult mammalian heart at single-cell rna-seq resolution
11	Chong J (2018) MetaboAnalyst 4.0: towards more transparent and integrative metabolomics analysis	9	Sayers, JR (2021) Heart regeneration: beyond new muscle and vessels
11	Virani SS (2020) Heart Disease and Stroke Statistics – 2020 Update:A Report From the American Heart Association	8	Dai, Z (2021) Recent progress in cardiovascular research involving single-cell omics approaches
10	Anderson JL (2017) Acute Myocardial Infarction	7	Xu, J (2020) Implications of gut microbiome on coronary artery disease
7	Tallquist MD (2017) Redefining the identity of cardiac fibroblasts	7	Qian, B (2022) Update on gut microbiota in cardiovascular diseases

In Cluster 11, which is titled “Artificial Intelligence,” the top 5 research bases are Baessler B (2018), Laroza A (2017), Ibanez B (2018), Oikonomou EK (2019), and Laroza A (2018). The top 5 research frontiers are Chang S (2022), Chong JH (2022), Willemink MJ (2021), Infante T (2021), and Jiang B (2020) (Table 7).

**Table 7:** Research base and research frontier of cluster 11

#11 Artificial intelligence			
Cited references		Citing references	
Freq	Author (year) title	Coverage	Author (year) title
16	Baessler B (2018) Subacute and Chronic Left Ventricular Myocardial Scar: Accuracy of Texture Analysis on Nonenhanced Cine MR Images	15	Chang, S (2022) Quality of science and reporting for radiomics in cardiac magnetic resonance imaging studies: a systematic review
14	Larroza A (2017) Differentiation between acute and chronic myocardial infarction by means of texture analysis of late gadolinium enhancement and cine cardiac magnetic resonance imaging	10	Chong, JH (2022) Artificial intelligence and cardiovascular magnetic resonance imaging in myocardial infarction patients
13	Ibanez B (2018) 2017 ESC Guidelines for the management of acute myocardial infarction in patients presenting with ST-segment elevation	9	Willemink, MJ (2021) Emerging methods for the characterization of ischemic heart disease: ultrafast doppler angiography, micro-ct, photon-counting ct, novel mri and pet techniques, and artificial intelligence
12	Oikonomou EK (2019) A novel machine learning-derived radiotranscriptomic signature of perivascular fat improves cardiac risk prediction using coronary CTangiography	8	Infante, T (2021) Radiogenomics and artificial intelligence approaches applied to cardiac computed tomography angiography and cardiac magnetic resonance for precision medicine in coronary heart disease: a systematic review
10	Larroza A (2018) Texture analysis of cardiac cine magnetic resonance imaging to detect non-viable segments in patients with chronic myocardial infarction	12	Jiang, B (2020) Development and application of artificial intelligence in cardiac imaging

## 4. Discussion

### 4.1. Analysis of production of publications

Figure 2 shows that omics research on myocardial infarction had fewer publications in the early phase, and the number of publications increased significantly in the last decade. The surge in literature can be attributed to several factors. Firstly, the continuous development and maturity of high-throughput techniques, along with the widespread adoption of omics concepts, have contributed to this phenomenon. Secondly, there has been an increase in the number of researchers engaged in all scientific research, leading to breakthroughs in specific areas. Overall, the output of scientific research has maintained an upward trend, aligning with the developmental patterns observed in general disciplines. It is important to note that the quantity of published papers does not solely reflect the quality and impact of the research, nevertheless, it indicates the popularity of the discipline. Considering the broad scope of omics research, the annual output of 145 papers in 2021 suggests that the discipline remains relatively unpopular and research gaps exist, signifying considerable potential for future development and research value.

### 4.2. Analysis of collaboration networks

Collaboration in scientific research plays a crucial role in promoting scientific advancement, enhancing research quality, and diversifying research topics.^[27,28]^ Collaboration can be classified into national, institutional, and author-level collaborations. In the form of national collaborations, institutions and authors are constituent elements, enabling the assessment of a country’s overall research strength. In the realm of myocardial infarction omics research, the United States leads in terms of both publications and the collaboration strength. Alongside active domestic collaborations, the United States has also demonstrated considerable involvement in international exchanges, indicating its prominent position in myocardial infarction research.

If the number of publications is high while overall collaboration intensity remains low, it would imply an inadequate knowledge mobility within the country’s research field, hindering the development of a robust academic ecosystem. Currently, most countries, except for the United States, exhibit low overall collaboration intensity, with most collaborations being domestic. It is suggest encouraging academic collaborations among authors and institutions involved in myocardial infarction research, particularly international.

As stable entities within the academic landscape, research institutions bear the responsibility of carrying out specific scientific research tasks. Inter-institutional collaborations generally offer greater stability and facilitate knowledge exchange. Additionally, since individual authors may belong to different institutions, collaborations among them can naturally enhance inter-institutional links. This study reveals that in the field of myocardial infarction, research institutions with close collaboration relationships are classified into the same cluster. Such clusters signify the coupling of research institutions through shared literature, suggesting that different institutions have overlapping or similar research directions. For instance, the yellow cluster in Figure 5 encompasses institutions such as Harvard University and Brigham Women’s Hospital, implying their alignment in the research focus. Given the specialized nature of myocardial infarction, research institutions predominantly concentrate within universities and medical institutions that renowned for their strong research capabilities and academic authority. Therefore, collaborating with these authoritative institutions is likely to enhance the professionalism and academic impact of myocardial infarction. Moreover, institutions within the same cluster can assist researchers in clarifying their specific research direction due to shared research content.

Compared to institutional collaboration, author collaboration forms more clusters, and the clustering tendency is more obvious. Author collaboration will make the literature co-authorship in a coupled state, although this state of cooperation is more loosely compared with institutional collaboration. The cooperation among authors is usually based on the academic background of interdisciplinary crossover, and the formation of academic groups based on a specific research topic. For example, the collaborative cluster led by Lindsey engages in extensive research on proteomics studies, including the analysis of matrix protease and extracellular matrix.^[29–31]^ Understanding author collaborations not only provides insights into the research content of different academic groups but also identifies valuable author clusters, facilitating communication and exchange among relevant author groups.

### 4.3. Words co-occurrence analysis

#### 4.3.1. Overview of co-occurrence words

The increasing number of keywords within the field of myocardial infarction omics is directly associated with the expanding literature output. The high retention rate of keywords and the limited number of newly added terms suggest a relatively conservative research approach in this area. Rather than pursuing broad horizontal expansion into new research directions, the current focus of myocardial infarction omics research appears to be on deepening existing knowledge. Nevertheless, as a concept with rich connotations, the study of myocardial infarction omics is still in its infancy and its branches hold tremendous research potential. Most of the existing terms serve as an essential foundation for the development of the discipline. The consistent increase in entropy of terms in the entropy map indicates the inclusion of more terms from different categories into the field, which suggests the trend of interdisciplinary research in the field of myocardial infarction omics will become increasingly prominent (Fig. 9).

#### 4.3.2. Three-fields plot

The three-fields plot has proven to be a valuable tool for identifying important research topics within the field of myocardial infarction omics. The important topics include Genomics, Metabolomics, Pharmacogenomics, Proteomics, and Lipidomics. In terms of paths, the number of paths flows from “genomics” to “reference literature” is the most (18 paths), while 11 paths flow from “author” to “genomics.” The majority of references flowing from “genomics” focus on the identification of risk loci through genome-wide association analysis.^[32,33]^

A key task in myocardial infarction genomics is to discover and verify risk loci leading to myocardial infarction. Based on the result of multi-omics cross-validation, functional annotation of existing risk loci can be made from a genomics perspective, which can be significant for identifying loci with similar or identical functions. Pharmacogenomics, which utilizes genomic information to study individual differences in drug response, holds promise in guiding clinicians toward precision personalized medicine. Metabolomics has the highest number of incoming flows in the three-fields plot, indicating the substantial interest of the influential author community in research on this topic. Notably, Lewis’s 2007 publication emerges as a significant flow in the literature of Metabolomics. This paper uniquely applies metabolomics to confirm changes in metabolite levels in myocardial infarction, thereby possessing important methodological and theoretical significance.^[34]^

In omics studies of myocardial infarction, a natural association exists between myocardial infarction and coronary heart disease, heart failure, and other related diseases. Consequently, the study of myocardial infarction omics is highly prevalent in research on acute coronary syndrome and other cardiovascular diseases.^[35,36]^ Thus, in addition to omics methods, cardiovascular disease and coronary artery disease are closely intertwined with myocardial infarction. There are many paths from these topics to reference literature, suggesting solid research bases in these areas. Moreover, topics such as atherosclerosis and inflammation, which are essential pathological processes in the occurrence of myocardial infarction and related diseases, have also become significant areas of research. These themes exhibit substantial overlap with the knowledge flow.

#### 4.3.3. Strategic diagram and evolution map

In this paper, the G-index is employed in SciMat to screen terms and create the Strategic Map and the Evolution Map. The G-index serves as a direct indicator of document quality and impact and can be used to assess the influence of the subject terms. The higher the G-index, the more influential the term.^[37]^

The strategic maps highlight differences strategic status of themes in different periods, while the evolution map can trace the evolution of different themes. This paper found 3 types of evolutionary paths according to the nature of themes.

##### 4.3.3.1. A path relating to myocardial infarction studies

*GENE-EXPRESSION SINGLE-NUCLEOTIDE-POLYMORPHISMS——ATHEROSCLEROSIS——GENOME-WIDE-ASSOCIATION*

This pathway represents a dynamic and evolving theme in the research on myocardial infarction omics, which is centered around genome-wide association analysis. The strategic map demonstrates the significant evolutionary potential of the *innovative themes,* GENE-EXPRESSION and SINGLE-NUCLEOTIDE-POLYMORPHISMS, which subsequently contribute to the emergence of the *engine theme*, ATHEROSCLEROSIS, in the subsequent period. ATHEROSCLEROSIS garnered substantial attention as a research hotspot from 2013 to 2017, eventually evolving into the broader theme, GENOME-WIDE-ASSOCIATION, from 2018 to 2022.

Genome-wide association analysis involves the identification of polymorphisms across the entire genome of multiple individuals, enabling the analysis of genotype-phenotype differences and the identification of genes most closely associated with the observed phenotype.^[38]^ In the context of myocardial infarction, researchers perform genetic tests on diverse populations and compare the genotypes of myocardial infarction patients and normal groups to identify candidate loci and relevant genetic variants. After lengthy and extensive studies, myocardial infarction susceptibility sites such as PCSK9 and APOB have been discovered using genome-wide association analysis.^[39,40]^ Importantly, these susceptibility sites often exhibit similar or identical pathological processes in terms of their expression. For instance, increased PCSK9 gene expression promotes the generation of LDL, while elevated APOC3 gene expression inhibits TG metabolism,^[41]^ both abnormal expressions predispose individuals to atherosclerosis, a significant contributor to myocardial infarction.

As a fundamental research method, genome-wide association analysis is expected to remain an ongoing *innovative theme* in the future, holding immense value for further investigation and exploration.

*PERCUTANEOUS CORONARY INTERVENTION PHARMACOGENETICS——CLOPIDOGRE, C-REACTIVE-PROTEIN——PHARMACOGENOMICS*

This pathway depicts the evolutionary progression of Pharmacogenomics in the context of myocardial infarction omics. The strategic diagram reveals that the *engine theme*, PERCUTANEOUS CORONARY INTERVENTION, PHARMACOGENOMICS, and the innovation theme, SINGLE-NUCLEOTIDE POLYMORPHISMS, has evolved into the engine theme, CLOPIDOGREL. Eventually, PHARMACOGENOMICS emerges after incorporating C-REACTIVE PROTEIN.

One influential achievement in myocardial infarction pharmacogenomics is the association between CYP2C19 gene polymorphism and cardiovascular disease. Early studies have demonstrated the significant clinical value of clopidogrel in the treatment of myocardial infarction and other cardiovascular conditions.^[42,43]^ Particularly for patients undergoing percutaneous coronary intervention (PCI), clopidogrel has been proven effective in reducing the occurrence of ischemic events.^[44]^ Based on these findings, researchers demonstrate that individuals carrying impaired CYP2C19 genes will experience disruptions in clopidogrel metabolism, leading to a higher incidence of adverse cardiovascular events.^[45]^ The effect is particularly pronounced in patients after PCI.^[46]^ The functional decline or loss of CYP2C19 inevitably affects the normal metabolic process of clopidogrel, resulting in diminished drug inhibitory effects and potentially leading to serious adverse cardiovascular events such as bleeding.^[47]^ In addition to CYP2C19, certain alleles of CYP2C9 and ABCB2 have also been found to influence clopidogrel metabolism.^[48,49]^

Pharmacogenomics is a crucial research approach in realizing precision medicine and individualized therapy. Currently, this theme occupies a pivotal position as an engine theme and holds significant research value.

##### 4.3.3.2. A path relating to physiological or pathological state

*OXIDATIVE-STRESS, ANTIOXIDANT——MICRORNAS, INFLAMMATORY-RESPONSE, BONE-MARROW, HEART——APOPTOSIS*

This pathway illustrates the evolutionary trajectory of the APOPTOSIS. The strategic diagram demonstrates that the integration of the *innovation themes* TOXIDATION-STRESS and ANTIOXIDANT led to the emergence of the other *innovation theme* MICRORNAS from 2013 to 2017, then, after absorbing the INFLAMMATORY-RESPONSE, BONE MARROW and HEART, the *engine theme* APOPTOSIS has formed.

Apoptosis refers to a spontaneous and programmed cellular death process occurring in normal physiological or pathological states.^[50]^ Presently, substantial evidence supports the association between apoptosis and oxidative stress mechanisms. When mitochondria undergos normal oxidative metabolism or when cells are exposed to abnormal environments, a significant amount of reactive oxygen species (ROS) is generated.^[51]^ When the accumulation of ROS surpasses the antioxidant capacity of cells, the body enters a state of oxidative stress.^[52]^ Under oxidative stress, ROS induces cell apoptosis by mediating various signaling pathways.^[53,54]^ When this process occurs within the cardiovascular system, it is likely to cause myocardial infarction and other adverse cardiovascular events.

Oxidative stress exerts its detrimental effects on the cardiovascular system in 2 primary ways. Firstly, it directly damages the vascular endothelial cell wall, leading to the accumulation and adhesion of inflammatory cells, thereby causing microcirculation dysfunction or atherosclerosis.^[55]^ Secondly, oxidative stress modulates the expression of relevant genes by influencing the level of transcription factors in the vascular wall, thereby aggravating the occurrence of cardiovascular diseases. From 2018 to 2022, apoptosis has emerged as a prominent topic in myocardial infarction omics research. As a significant physiological process, it holds the potential for future integration with omics studies, such as transcriptomics, to explain the mechanism of adverse cardiovascular events.

##### 4.3.3.3. A path relating to a biological structure

*ACUTE-CORONARY-SYNDROME——QUANTITATIVE-PROTEOMICS——CARDIAC-FIBROBLASTS*

This pathway describes the evolution of the study of cardiac fibroblasts and its significance in various stages. The strategic diagram illustrates the progression from ACUTE-CORONARY SYNDROME to QUANTITATIVE-PROTEOMICS, and ultimately to CARDIAC-FIBROBLASTS. Following myocardial infarction, Cardiac-Fibroblasts assume a critical role in myocardial scar repair.^[56]^ Meanwhile cardiac fibroblasts are closely associated with ventricular remodeling and post-infarction heart failure.^[57,58]^ Scholars have investigated signaling pathways linked to this theme using diverse omics methodologies, such as genome, transcriptome, and proteome, confirming the involvement of inflammatory cytokines TGF-β,^[59,60]^ RAAS,^[61]^ several miRNAs,^[62,63]^ and other regulatory factors in cardiac fibroblast regulation. Despite the current positioning of this theme at the junction of the second and third quadrants, it still holds substantial research value and with the potential to evolve into an engine theme in the future.

*SINGLE-NUCLEOTIDE-POLYMORPHISMS, STROKE——EPIDEMIOLOGY, FATTY-ACIDS, QUANTITATIVE-PROTEOMICS, LIPOPROTEINS, C-REACTIVE-PROTEIN——CHOLESTEROL*

This paragraph discusses the evolutionary path of cholesterol as a research theme and emphasizes its importance in human tissues and cells. The strategic diagram indicates that the themes of SINGLE-NUCLEOTIDE-POLYMORPHISMS and STROKE converge into the *engine theme* of EPIDEMIOLOGY. Through the integration of FATTY-ACIDS, QUANTITATIVE-PROTEOMICS, LIPOPROTEINS, and C-REACTIVE-PROTEIN, the theme further evolves into CHOLESTEROL. Cholesterol is a vital substance with indispensable roles in human tissue cells. It comprises 2 primary types, HDL-C and LDL-C, both of which significantly influence the development and outcome of cardiovascular and cerebrovascular diseases. HDL-C is commonly believed in protect blood vessels, while LDL-C can contribute to atherosclerosis and thrombosis formation.^[64]^ Researchers commonly employ proteomic methods to quantitatively analyze lipoproteins and elucidate the biological functions of specific lipoprotein subtypes.^[65,66]^ In the study of metabolomics, scholars conduct quantitative investigations in the form of lipidomics by observing metabolic changes of cholesterol after myocardial infarction to identify novel biomarkers in lipid metabolism.^[67]^ Meanwhile, researchers have explored the mechanisms of lipid molecules during myocardial infarction.^[67,68]^ Genomics studies have also demonstrated that the variation of KLOTHO gene and 1p13.3 gene is closely associated with the levels of HDL-C and LDL-C,^[69,70]^ successfully establishing a link between cholesterol and myocardial infarction. Currently, CHOLESTEROL represents an *innovative theme* and a popular research direction, and it will undoubtedly remain a prominent research area in the future.

#### 4.3.4. Analysis of co-occurrence words network

The clustering of the co-occurrence words network yields a co-occurrence word clustering map and a timeline map (Figs. 11 and 12) The clustering map provides an overview of theme clustering, while the timeline map illustrates the temporal distribution within the cluster, highlighting burst terms and centrality terms (Fig. 13; Table 1).

The identification of burst and centrality terms holds great importance as it enables researchers to identify significant research topics both in the past and the present. In the co-occurrence network, burst words and centrality terms are assigned higher weights. Burst words represent topics that have received substantial attention within a specific timeframe, while centrality terms represent subjects of important research significance in the construction of the whole research theme.

According to the co-occurrence words network, burst terms predominantly appear within cluster 3 (Coronary Art Disease), cluster 4 (Heart Failure), and cluster 9 (Genetics). This suggests that these clusters have ever already produced significant research topics. On the other hand, centrality terms are widely distributed across multiple clusters, indicating their relevance and connection to various research areas.

##### 4.3.4.1. Cluster#0 Extra Cellular Matrix

Cluster 0 focuses on the study of Extra Cellular Matrix. During myocardial infarction, cardiac fibroblasts secrete a large amount of extracellular matrix, which plays a vital role in the repair of cardiac tissue.^[71,72]^ There is a burst in this cluster, left ventricle, which corresponds to the study on left ventricular remodeling after myocardial infarction. A prominent research topic within this cluster is matrix metalloproteinases (MMPs), which is crucial regulatory factors of extracellular matrix components. MMPs are involved in the degradation of extracellular matrix,^[73]^ which is closely associated with left ventricular remodeling after myocardial infarction and often impacting the outcome of the condition.^[29,74]^ For instance, studies have demonstrated that MMP-9 can regulate myocardial tissue remodeling by activating cytokines and chemokines, serving as an important inducer affecting left ventricular remodeling.^[75]^ On the other hand, MMP-12 can inhibit neutrophil apoptosis, thereby affecting the normal remodeling function of the left ventricle.^[30]^ Some researchers suggest that a detailed understanding of the role of MMPs could potentially lead to the aimful inhibition or promotion of relevant targets, thus, effectively treating myocardial infarction.^[71]^ Furthermore, aside from studies on matrix metalloproteinases, other intriguing topics in this cluster, such as cardiac fibroblasts, are closely related to cardiac remodeling and fibrosis processes.

##### 4.3.4.2. Cluster #1: Pharmacogenomics

Cluster 1 focuses on the field of pharmacogenomics, encompassing themes with high centrality such as mass-spectrometry, heart disease, acute myocardial infarction, bioinformatic analysis, and burst theme such as proteomic analysis. The concentrated presence of these important themes indicates the significant role of pharmacogenomics in myocardial infarction research. A prominent research theme within this cluster is CYP2C19 gene polymorphism. Clopidogrel therapy, an anti-platelet therapy, has shown significant efficacy in reducing the severity of atherosclerosis. However, not all patients are eligible for this therapy, and anti-platelet drugs like clopidogrel should be avoided if the patient’s genotyping suggests a loss-of-function phenotype.^[76,77]^ Furthermore, researchers are actively investigating the impact of individual genes on the effectiveness of alternative drugs. Studies have also highlighted the need for different anticoagulant therapy strategies based on gene variants of CYP2C9,^[78]^ VKORC1,^[79]^ and CYP4F2.^[80]^

##### 4.3.4.3. Cluster #3: Coronary Artery Disease

Cluster 3 centers around the study of coronary artery disease. The narrowing and obstruction of arteries due to coronary artery disease can lead to reduced blood supply to the myocardium, which is considered a major cause of myocardial infarction.^[81]^ Coronary artery disease and myocardial infarction are pathologically related, and their co-occurrence frequency has significantly increased recently. Academic studies on coronary artery disease have been ongoing for a considerable period. In earlier time periods, research primarily focused on the metabolism of coronary artery disease and related conditions.^[82,83]^ Subsequently, studies explored biomarkers and targeted therapies for cardiovascular diseases.^[84,85]^ Recent investigations have primarily focused on endothelial cells,^[86]^ high-density lipoprotein,^[87]^ and low-density lipoprotein.^[88]^ This cluster encompasses a rich array of research themes, and its content is expected to become increasingly valuable as omics studies continue to advance.

##### 4.3.4.4. Cluster #4: Heart Failure

Cluster 4 pertains to studies on heart failure. Heart failure is not a standalone disease but rather a common outcome of various heart conditions. It often occurs following myocardial infarction and is closely linked to ventricular remodeling. Consequently, future omics studies on heart failure may emphasize preventive measures. The burst terms within this cluster indicate a research focus on genetic polymorphisms in heart failure and drug therapy. Genomics studies have identified the 2 susceptible risk loci of “all-cause HF” and “non-ischemic HF.” Although ischemic heart disease risk loci can undoubtedly cause heart failure, identifying non-ischemic HF risk loci is crucial from a genetic perspective for prevention and treatment of heart failure.^[89]^

##### 4.3.4.5. Cluster #9: Genetics

Cluster 9 focus on the genetics of myocardial infarction and related diseases. Themes of “genetic factor,” “genetic variants,” and “genome wide association study” within this cluster demonstrate high burst characteristics. The high centrality of “genome-wide association study” indicates its significance as a research hotspot. Studies conducted within this cluster have investigated susceptible loci and variant genes associated with myocardial infarction and related diseases.^[90–92]^ With the maturation of genome-wide association study (GWAS) technology and the decreasing costs of research, the number of risk loci linked to myocardial infarction and related diseases has continued to grow. Starting from the early-stage study of the 9p21 site,^[32,33]^ there are now over 160 sites and more than 300 candidate genes that are considered to have a significant correlation with myocardial infarction and related diseases.^[91]^ The study of large databases is likely to become an important research topic within this cluster. In the future, risk gene databases will serve as essential foundations for clinicians to diagnose myocardial infarction and related diseases. With outcomes of different omics studies, the diagnostic capabilities of risk gene databases will be further enhanced, providing significant practical value in identifying relevant etiologies, pathogenesis, and targeted and precise treatments. Genetics remains a prominent and ongoing topic.

In addition to the discussed clusters, other themes such as Myocardial Infarction, STEMI, Artificial Intelligence, and Gut Microbiota show excellent development trends. These clusters contribute to the overall profile of myocardial infarction omics. Clusters of Artificial Intelligence and Gut Microbiota provide alternative perspectives and research directions. For example, AI technology assists physicians in making judgments on cardiac imaging,^[93,94]^ while machine learning technology helps identify biomarkers of myocardial infarction and explores the role of intestinal colonies in the process of atherosclerosis.^[95]^ The regulation of Gut Microbiota is also being investigated to prevent the occurrence of atherosclerosis.^[96]^ These clusters offer unique insights and research directions for studying myocardial infarction patterns.

### 4.4. Citing and cited journal analysis

Journals play a crucial role as a medium for researchers to acquire knowledge in their respective fields. Depending on their specific needs, researchers select relevant cited journals and citing journals to obtain both foundational and cutting-edge research information. Figure 19 illustrates that most of the cited journals are authoritative publications, including *NATURE, NEW ENGLAND JOURNAL OF MEDICINE, CIRCULATION*, and others. These journals serve as the primary sources for the literature that build the foundation of the field. On the other hand, researchers should particularly focus on the citing journals. These journals publish articles at the forefront of research in the field and are directly relevant to the observational study of myocardial infarction. They serve as valuable references for researchers’ own contributions.

In terms of knowledge flow, there is no significant difference in the direction between cited journals and citing journals. (Fig. 20) The distribution of disciplines in research base and research frontier also shows no significant difference. However, the research base tends to be oriented towards practical disciplines such as nursing and clinical studies. In contrast, the research frontiers are more inclined towards theoretical disciplines such as molecular biology and immunology. Nevertheless, the overall research is concentrated in the biomedical field. Additionally, other disciplines like mathematics, physics, and computer science have emerged as research bases, bringing interdisciplinary perspectives to the forefront of myocardial infarction omics. The integration of multiple disciplines has promoted interdisciplinary interactions within the field.

### 4.5. Co-citation and clustering analysis

#### 4.5.1. Analysis of centrality

Centrality analysis of documents helps identify papers that are widely recognized and influential within the academic community. A high degree of centrality indicates that the research theory or results have garnered significant attention and the paper itself is extensively cited.^[97]^

The results of centrality analysis reveal that the most central paper, published by Schunkert H in NATURE GENETICS in 2011, achieved a centrality score of 0.27. This study conducted a large-scale genome-wide correlation analysis and confirmed 13 susceptible loci related to coronary heart disease. The findings provided new insights into the pathogenesis and the prevention of coronary heart disease from a genomic perspective.^[98]^ The loci identified in this study has been repeatedly verified by subsequent studies. For example, the 9q34.2 locus, associated with the ABO blood group, has been directly linked to the incidence of myocardial infarction in subsequent research.^[90]^

The second most central paper, published by Deloukas P in NATURE GENETICS with a centrality score of 0.24, employed whole-gene association analysis to identify new susceptibility loci associated with coronary heart disease, expanding the number of susceptibility loci to 46. Additionally, the study analyzed gene expression interaction networks and identified important pathways related to lipid metabolism and inflammatory response, providing insights into the risk factors leading to myocardial infarction in terms of genetic pathways.^[40]^

In 2017, Benjamin EJ et al from the American Heart Association, published a report on heart disease and stroke in Circulation.^[64]^ This report offers a wealth of fundamental data commonly cited by other studies.

In addition to the above-mentioned papers, other highly central articles focus on genetic studies of myocardial infarction and related diseases. For instance, ADAMTS7 has been identified as a risk locus for coronary heart disease and has been shown to induce atherosclerosis through mouse experiments.^[99]^

The findings from the centrality analysis of these literature sources reinforce the crucial role of genomics in the study of myocardial infarction.

#### 4.5.2. Analysis of burst references

High burst literature refers to scientific papers that receive a high number of citations within a short period, typically indicating significant scientific breakthroughs or discoveries with important academic implications.^[100]^ It is possible that a literature has both high centrality and high burst values. However, the high burst emphasizes the timeliness of the citations, indicating a surge in attention during a specific time interval. In the burst timeline diagram, the start and end points represent the beginning and the end of the burst period. It’s important to note that after the end point, the literature is not no longer referenced but not cited frequently. The strength of the burst is proportional to the number of citations within that interval, and researchers can refer to relevant burst literature as needed. High burst literature is generally considered as a hot research outcome, while the recent high burst literature represents future research trends.

The results of the burst analysis reveal that the first burst occurred between 2004 and 2009, indicating increased attention of that literature during 6 years. Through genome-wide association analysis, the study confirmed that mutations in ALOX5AP, which encodes the FLAP gene, increase leukotriene production in arterial walls, leading to inflammatory responses and the development of myocardial infarction and stroke.^[101]^ Recent burst literature includes the case data of heart disease and stroke published by Go AS in 2014,^[102]^ and the application of proteomics in cardiovascular diseases discussed by Lindsey ML in 2015.^[103]^ The former refers to the reporting of disease-related data, which is widely cited due to providing basic information such as mortality and incidence rates of the respective diseases. It should be noted that such data reports will be updated continuously and has a with strong timeliness. The latter discusses the comprehensive proteomics system for cardiovascular disease, covering standardized study designs, experimental method comparisons, network analysis methods, and so on. It provides guidance for subsequent researchers to conduct proteomics experiments. Other burst literature is mainly related to genomics. For example, the highest burst literature (15.34) was published by Samani NJ in the NEW ENGLAND JOURNAL OF MEDICINE in 2007, where the authors identified loci significantly associated with coronary artery disease by searching for repeated loci.^[33]^

These burst analysis results once again underscore the crucial role of genomics in the study of myocardial infarction omics. When researchers encounter literature with high burst, they can try to associate it with clusters and interpret the results from the perspective of parallel clusters.

#### 4.5.3. Analysis of clustering

Using the clustering function of Citespace, this paper clusters all the cited references obtain a total of 11 main clusters. The Silhouette values for all clusters are high, indicating favorable classification results. The results of cited papers roughly reflect the research bases of myocardial infarction omics, while the citing papers represent important and influential results within specific research directions.

In this paper, 4 clusters were selected for their representative significance.

Cluster 0, labeled “coronary artery disease,” likely encompasses a comprehensive body of literature related to this disease. It is characterized by high centrality and high burst, indicating that it contains influential and highly cited papers in the direction of coronary artery disease. This cluster is of considerable importance in the field.

Cluster 1, labeled “individual variability,” focuses on the variability observed in individuals concerning myocardial infarction. This cluster also exhibits high centrality and high burst, suggesting that it contains seminal works that have attracted significant attention. Understanding individual variability can help in personalizing treatment approaches and improving patient outcomes.

Cluster 2, labeled “gut microbiome,” represents a recent research hotspot in the field of myocardial infarction. This cluster likely includes studies that investigate the role of the gut microbiome in the development, progression, and treatment of myocardial infarction. The inclusion of this cluster indicates the growing interest in the interaction between the gut microbiome and cardiovascular health.

Cluster 11, labeled “artificial intelligence,” highlights the emerging trend of using AI techniques in myocardial infarction omics. This cluster may cover studies that utilize AI for tasks such as image analysis, predictive modeling, or data mining in the context of myocardial infarction. AI has the potential to revolutionize diagnosis, treatment, and prevention strategies for myocardial infarction.

By examining these selected clusters, researchers can gain insights into the foundational literature, recent research trends, and emerging areas of interest in the study of myocardial infarction omics.

##### 4.5.3.1. Cluster#0 Coronary Artery Disease

###### 4.5.3.1.1. Research base

The clustering analysis in this study has provided insights into the relationship between coronary heart disease and myocardial infarction. The first cited literature in the cluster, Lloyd-Jones DM’s review in 2004, focused on risk factors for cardiovascular disease and validated genetic risk factors that predict the likelihood of disease in offspring.^[104]^ Since then, genetic research has played a significant role in exploring the pathogenesis of coronary heart disease.

Among the literature with high centrality, Frazer Ka’s study described the Phase II HapMap, which collected a vast amount of genetic data from millions of individuals.^[105]^ This research contributed to the understanding of genetic variation in different populations. Anderson JL conducted pharmacogenetic studies on the application of warfarin in anticoagulant patients, demonstrating the potential of pharmacogenetics in improving the efficiency and accuracy of drug administration.^[106]^

The highly burst literature in the cluster also reflects the emphasis on genetic research. Both McPherson R’s review of the function of chromosome 9 alleles associated with coronary heart disease and Kathiresan S’s search for risk loci associated with early-onset myocardial infarction through whole-gene association analysis are examples of genetic studies that have received significant attention.^[32–39]^ These studies have enriched the field of myocardial infarction genomics.

Overall, the clustering analysis highlights the central role of genetics in the study of myocardial infarction, as evidenced by the foundational, high centrality, and highly burst literature in this cluster. Genetic research has provided valuable insights into the pathogenesis and risk factors of coronary heart disease and myocardial infarction omics, contributing to advancements in the field of genomics.

###### 4.5.3.1.2. Research frontier

The research frontier of this disease builds upon the research bases and continues the exploration of genomics. This section mainly focuses on targeted genome screening, indicating the standardization and maturity of genomics research. To enhance collaboration and increase the chances of discovering risk loci for coronary heart disease and myocardial infarction, the Coronary Artery Disease Genome-wide Replication and Meta-analysis (CARDIoGRAM) consortium was formed.^[107]^ Subsequent studies within the consortium have not only identified an increasing number of risk loci but have also confirmed that mutations at certain loci can contribute to the development of different cardiovascular diseases.

Building upon the identification of risk loci for coronary heart disease, many researchers have expanded their investigations into other areas within the field of cardiovascular disease.^[108,109]^ This broader exploration is crucial for establishing a comprehensive annotation of genomic loci and understanding their implications across various cardiovascular conditions. While this clustering analysis specifically focuses on the clustering of coronary heart disease within the context of myocardial infarction studies, it is important to note that advancing research on coronary heart disease requires interdisciplinary collaboration and investigations from multiple disciplines.

In summary, while genetic research has played a significant role in understanding coronary heart disease and myocardial infarction, the research frontier acknowledges the need for multidisciplinary approaches to advance the understanding of these conditions. The formation of consortiums, the identification of risk loci, and the expansion of research into other cardiovascular areas contribute to the comprehensive exploration of genomics and its implications in cardiovascular disease research.

##### 4.5.3.2. Cluster#1 Individual Variability

###### 4.5.3.2.1. Research base

In this cluster, the focus is on individual differences in response to treatment, particularly in the context of clopidogrel. The aforementioned study on cardiovascular events in CYP2C19 variant carriers treated with clopidogrel is a highly influential literature within this cluster.^[45]^ Most of the studies in this cluster are centered around pharmacological explorations of clopidogrel, including the validation of therapeutic impact of novel CYP2C19 genotype variants and the identification of genotypes that contribute to individual differences in treatment response.

Shuldiner AR demonstrated that CYP2C19*2 variants can result in reduced therapeutic efficacy of clopidogrel, leading to adverse cardiovascular outcomes.^[110]^ Collet JP also conducted cohort tests confirming that CYP2C19*2 variants were the major determinant of outcomes in patients treated with clopidogrel.^[111]^ Bouman HJ found that the Q192R variation of the PON1 enzyme was a primary determinant of clopidogrel efficacy, although this view was later questioned by Lewis et al.^[112,113]^ Some scholars approached the topic from a pharmaceutical perspective, seeking additional therapeutic agents that could account for individual differences. Holmes MV demonstrated that the efficacy of clopidogrel was not related to the polymorphism of CYP2C19 and ABCB1.^[114]^ Clustering, with a focus on individual differences in drug treatment, has led to a substantial body of research on pharmacogenomics.

###### 4.5.3.2.2. Research frontier

In the research frontier cluster, the literature continues to focus on pharmacogenomics, further expanding the scope of myocardial infarction research. Compared to earlier studies, the literature in this part delves deeper and explores more specific aspects, including multi-pathway verification of gene variations affecting drug therapeutic effects and mechanism research on gene variations influencing drug responses.^[115]^ In the search for new genetic variations, Lewis found that the genetic variation of CES1 may be associated with clopidogrel treatment.^[116]^ The ultimate goal of these studies is to guide clinical personalized medicine and effectively improve patient survival.

##### 4.5.3.3. Cluster#2 Gut Microbiome

###### 4.5.3.3.1. Research base

The gut microbiome has indeed emerged as a recent research hotspot in the field of myocardial infarction and cardiovascular disease. In the co-occurrence word analysis clustering, a specific cluster focuses on exploring the relevance of microbial communities in the gut to the immune, metabolic, and hormonal properties of the host. This cluster represents the beginning of research into the role of the gut microbiome in myocardial infarction, and its important research bases lie in understanding the mechanisms of disease within the single-cell communities of endothelial cells.

Within this cluster, studies have examined various aspects such as the entire process of inflammation, fibrosis, repair, and regeneration,^[117]^ as well as the analysis of T-cell and macrophage characteristics in carotid plaque.^[118]^ Additionally, comprehensive integration of single-cell transcriptional profiles has provided insights into the role of microbial communities in myocardial infarction.^[119]^

In recent literature, there has been a growing focus on the study and application of microbial populations in cardiovascular disease. For example, Jie conducted a comparative analysis of gut microbiota data from different atherosclerotic cardiovascular diseases, identifying distinct microbial populations associated with specific diseases.^[120]^ This research provides novel insights into the prevention of arterial diseases and related conditions.

Edward S. Chambers assessed the impact of short-chain fatty acids produced by gut microorganisms on the heart.^[121]^ Zhu W.F. revealed the mechanisms of thrombosis, and that new gut microbial flora promotes platelet function.^[122]^ These studies primarily investigate the metabolic pathways of microbial populations and their relevance to myocardial infarction. By studying microbial populations, researchers can gain valuable insights into the metabolic aspects of myocardial infarction.

###### 4.5.3.3.2. Research frontier

Indeed, the articles within this cluster can be categorized into 3 directions: single-cell studies, metabolomics studies, and gut microbiome studies. These areas of research are interconnected and contribute to a comprehensive understanding of myocardial infarction.

Single-cell studies focus on exploring the communication pathways between cells based on their heterogeneity and establishing cell maps of related diseases.^[123]^ For example, Thankam utilized single-cell RNA sequencing (scRNAseq) to simulate myocardial ischemia-reperfusion and identified subpopulations of epicardial adipose-derived stem cells, highlighting their role in cardiac repair.^[124]^ Wu, L characterized the single-cell transcription profiles of mouse’ left ventricular cell landscapes using single-cell RNA sequencing, revealing extensive intercellular communication networks within the left ventricle.^[125]^ Single-cell RNA sequencing serves as a crucial bridge connecting microbiome research, and with the widespread application of microbial high-throughput single-cell sequencing technology, microbiome research is expected to enter a new era of investigation.^[126]^

In specific studies on gut microbiome, researchers have confirmed the protective role of certain microbial metabolites in myocardial infarction. Zhou’s study demonstrated that 3-HPA and 4-HBA derived from the gut microbiome can play a protective role in myocardial infarction. Xu’s study identified the composition of gut microbiome in patients with coronary artery disease and analyzed its potential mechanisms in the pathogenesis of coronary artery disease. The prognostic value of gut microbiome in coronary artery disease is also emphasized.^[127]^ The emerging evidence consistently highlights the crucial role of intestinal microbial community in cardiovascular diseases.^[128]^ It is foreseeable that further investigations into gut microbes may lead to new breakthroughs in the study of myocardial infarction.

##### 4.5.3.4. Cluster#11 Artificial Intelligence

###### 4.5.3.4.1. Research base

The recent advancements in AI have significantly influenced various fields, including medicine, fostering their rapid development. Within the realm of medicine, AI has emerged as a pivotal tool in myocardial infarction omics studies, and noteworthy contributions can be found in the literature. For instance, Baessler B effectively employed texture analysis to accurately diagnose subacute and chronic myocardial infarction through non-enhanced magnetic resonance imaging.^[129]^ Similarly, Larroza A utilized machine learning techniques to analyze texture features extracted from magnetic resonance imaging, thereby enabling the distinction between acute and chronic myocardial infarction.^[130]^ Subsequent research successfully employed texture analysis to differentiate between involved non-viable, viable, and remote segments in cardiac magnetic resonance imaging.^[131]^ The consistent application of texture analysis across these studies underscores its significance as a fundamental research method in the context of myocardial infarction investigations leveraging AI. Texture analysis entails an image analysis technique that quantifies variations in image intensity. Particularly within the realm of biomedical imaging, texture analysis assumes a crucial role as an algorithmic feature within imaging omics, facilitating the digital interpretation of medical images.^[132]^ Texture analysis, being a central concept in computer vision, is poised to drive the future development of imaging as it becomes increasingly integrated with deep learning. Furthermore, Oikonomou EK employed machine learning to analyze the radiological characteristics of Perivascular adipose tissue surrounding coronary arteries, thereby predicting the risk of heart disease.^[133]^ The pursuit of disease imaging biomarkers guided by AI technology holds immense potential to revolutionize future diagnostic models, making the exploration of such AI-driven diagnostic models highly promising and worthy of research. However, it is important to note that AI application in cardiology has only recently gained momentum, signifying substantial room for growth and future prospects.

###### 4.5.3.4.2. Research frontier

A number of scholars have conducted comprehensive reviews on the studies pertaining to AI in myocardial infarction omics.^[134,135]^ Moreover, building upon the ideas put forth by Oikonomou EK, Si et al developed a combined model incorporating the perivascular fat attenuation index and peri-coronary adipose tissue, demonstrating exceptional diagnostic capabilities.^[136]^ Similarly, Lin A constructed a joint recognition model utilizing machine learning that incorporated attenuation data, imaging parameters, and clinical features of peri-coronary adipose tissue, yielding positive outcomes in the diagnosis of myocardial infarction.^[137]^ Avard devised a machine learning algorithm specifically tailored to discern myocardial infarction in non-enhanced cardiac MRI.^[138]^ Currently, the predominant focus of myocardial infarction omics research lies in the domain of image-based AI applications. Machine learning techniques, particularly deep learning, are employed to extract pertinent features and train predictive models for myocardial infarction diagnosis. Nonetheless, the utilization of AI algorithms in clinical practice is currently impeded by concerns regarding security, privacy, ethics, and other associated risks. Consequently, the existing diagnostic models constructed by AI algorithms can only be employed as complementary tools for clinicians, necessitating further interdisciplinary collaborative research before they can be effectively applied in clinical diagnostic practices.

Due to space constraints, a comprehensive analysis of clustering cannot be undertaken within the scope of this paper. Apart from the clustering approach discussed in this article, myocardial infarction omics research encompasses investigations into miRNAs relevant to the transcriptome,^[139]^ analyses of inflammatory networks associated with myocardial infarction metabolic pathways,^[140]^ and explorations of myocardial infarction omics from the perspective of traditional Chinese medicine.^[141]^ In essence, the findings derived from co-citation analysis and co-word analysis of the literature effectively complement and substantiate one another.

## 5. Conclusion

Omics research provides a holistic and systematic perspective for studying disease occurrence and outcomes. As a significant health concern worldwide, myocardial infarction holds immense research value. And omics research based on interdisciplinary knowledge provides a broader scope for traditional myocardial infarction research.

In this study, the authors apply a bibliometric approach to collect literature data on myocardial infarction omics research in a more comprehensive way with a global perspective and use the literature data as the object of analysis, combined with bibliometric visual scientific mapping analysis tools to show the complete development history of myocardial infarction omics research. By depicting the knowledge structure in this field, the current research landscape is outlined, along with guidance and suggestions for subsequent omics research.

In terms of the overall intellectual framework, this paper can be divided into 4 parts. Firstly, it presents the collaborative landscape of omics research on myocardial infarction at 3 levels: country, institution, and author, which emphasize the conceptual differentiation of these cooperative relationships and provides targeted guidance and recommendations from the perspectives of institutions and authors. This point has never been mentioned in previous bibliometric articles. Secondly, a detailed analysis of research shifts in the field of myocardial infarction pathology are conducted. Specifically, the distribution of thematic terms indicates significant potential for future research in this area; the three-field-plot reflects the research content and knowledge flow paths of the field; the evolution map reflects the evolution history of the terms. This paper proposes 3 types of partition according to the nature of the path, which are evolutionary path of research methods, path of physiological or pathological states, and path of biological structures. Representative terms under each partition are identified and analyzed with the consideration of their evolution in strategic coordinates. Topic network mapping presents a more comprehensive picture of the linkage of terms under this research area, and after clustering the terms, representative clusters are selected for discussion. In the third part of the paper, the relationship between cited and citing journals is analyzed to clarify the knowledge flow between research bases and research frontiers. Lastly, a co-citation analysis is conducted, focusing on highly centered literature and literature with citation burst. The current research hotspots are discussed through clustering analysis, considering both research bases and research frontiers. However, there are still some shortcomings in this paper. Given its length, the intricacy of integrating multiple bibliometric tools, and the substantial volume of literature involved, results or paths with representative significance were selectively chosen for a limited interpretation. Consequently, not all results were comprehensively analyzed and explained. Nevertheless, all the atlas data were presented for researchers who are interested in further investigations.

To summarize, collaboration in myocardial infarction omics studies is still in its early stage, with limited international exchange and cooperation. Researchers in this field can enhance communication by fostering institutional mobility. Given the unique nature of omics research, authors from different disciplinary backgrounds are encouraged to collaborate. The flow of knowledge between disciplines does not exhibit significant differences. Although the field is currently in a relatively conservative state, there are non-biomedical disciplines joining the knowledge flow, indicating a trend toward diverse interdisciplinary integration. Genomics remains a major research topic in the field, but progress is also being made in other omics research areas. With the advent of AI and the big data era, emerging directions will be integrated into omics research of myocardial infarction, expanding the research methods and capabilities of omics for myocardial infarction and related diseases. Ultimately, this will facilitate personalized treatment and improve prognoses for myocardial infarction patients.

## Author contributions

**Conceptualization:** Yue Zhong, Tiantong Yang.

**Data curation:** Min Wang, Zhengqi Han, Tiantong Yang.

**Formal analysis:** Zhengqi Han, Mengzhou Zhang, Tiantong Yang.

**Funding acquisition:** Tiantong Yang.

**Investigation:** Zhengqi Han, Yue Zhong, Mengzhou Zhang.

**Methodology:** Zhengqi Han, Yue Zhong.

**Project administration:** Yue Zhong, Tiantong Yang.

**Resources:** Min Wang, Tiantong Yang.

**Software:** Xuan Wei, Min Wang, Chang Li.

**Supervision:** Min Wang, Shengnan Yu, Chang Li.

**Validation:** Xuan Wei, Min Wang, Shengnan Yu, Chang Li.

**Visualization:** Xuan Wei, Chang Li.

**Writing – original draft:** Xuan Wei.

**Writing – review & editing:** Xuan Wei, Shengnan Yu.
